# Delta-like ligand 4 mediated myeloid-derived suppressor cell metabolic reprogramming promotes neoadjuvant therapy resistance in titin-inactivated triple-negative breast cancer

**DOI:** 10.1186/s43556-025-00372-6

**Published:** 2025-12-02

**Authors:** Yanfang Yang, Ziyun Liu, Parhat Kaysar, Yuxi Han, Bo Ni, Linwei Li, Lina Zhang, Xiaobin Shang, Yaoyao Zhou, Yongjie Xie, Zhansheng Jiang

**Affiliations:** 1 Key Laboratory of Cancer Prevention and Therapy, Tianjin, Tianjin’s Clinical Research Center for Cancer, Tianjin Medical University Cancer Institute and Hospital, National Clinical Research Center for Cancer, Tianjin, 300060 China; 2 The Second Surgical Department of Breast Cancer, Tianjin Medical University Cancer Institute and Hospital, Tianjin, 300060 China; 3 Pancreas Center, State Key Laboratory of Drug Ability Evaluation and Systematic Translational Medicine, Tianjin Key Laboratory of Digestive Cancer, Tianjin’s Clinical Research Center for Cancer, Tianjin Medical University Cancer Institute and Hospital, National Clinical Research Center for Cancer, Tianjin, P. R. China; 4 Department of Thoracic Surgery, National Cancer Center/National Clinical Research Center for Cancer/Cancer Hospital, Chinese Academy of Medical Sciences and Peking Union Medical College, Beijing, China; 5 Department of Integrative Oncology, Tianjin Medical University Cancer Institute and Hospital, Tianjin, 300060 China

**Keywords:** TNBC, TTN inactivation, MDSCs, Glycolytic metabolism, DLL4-MCT4 axis, Immune escape

## Abstract

Triple-negative breast cancer (TNBC) is characterized by complex genomic background and treatment resistance. We first defined “Titin (TTN) inactivation”, a state of TTN expression deficiency or mutation, affecting tumor progression. However, how TTN inactivation regulates immune escape and affects therapeutic resistance remains unclear. Using whole-exome sequencing, single-cell transcriptome sequencing, and spatial transcriptome sequencing, we screened the clinical features of TNBC patients with TTN inactivation who accepted neoadjuvant therapy. Meanwhile, we used CRISPR-Cas9 technology to construct various mutant TNBC cell lines. Lentiviral vector carrying TTN and delta-like ligand 4 (DLL4) was validated in vivo to verify potential mechanism. Myeloid-derived suppressor cells (MDSCs) metabolic function was measured using glycolysis-related molecular experiments. Immunotherapeutic agents against TNBC with TTN inactivation were explored in the orthotopic MCT4^fl/fl^ genetically modified mouse model. DLL4-regulated ecological niche was established in TNBC with TTN inactivation. Mechanistically, TTN deficiency and mutation led to DLL4 secretion in TNBC. DLL4 enhanced MCT4-mediated glycolysis via MDSCs-derived NOTCH2 signaling pathway, driving the malignant function and lactate acid excretion of MDSCs. DLL4-derived MDSCs promoted stemness-mediated drug resistance by inducing histone lactate modification in TNBC, suppressing the anti-tumor activities of CD8^+^T cells. Blocking the DLL4-MCT4 axis stimulated anti-tumor immunity and synergized with anti-PD-1, improving response rates for first-line neoadjuvant therapy in TNBC. Our study revealed intrinsic mechanism by which TTN regulates the tumor immune microenvironment and provided a potential target for immunotherapy in TNBC with TTN inactivation.

**Supplementary Information:**

The online version contains supplementary material available at 10.1186/s43556-025-00372-6.

## Introduction

The global incidence of triple-negative breast cancer (TNBC) has been increasing, accounting for 15%–20% of breast cancer cases. TNBC is aggressive and associated with poor prognosis due to its high metastatic and recurrence rates, as well as resistance to conventional chemotherapy and immunotherapy [1]. Current treatments, such as Nab-PTX, show limited efficacy and considerable toxicity [2]. Although genomic studies have advanced our understanding of TNBC, the mechanisms underlying treatment resistance and immune evasion remain unclear.

Genomic alterations in TNBC, particularly their impact on response to chemotherapy and immunotherapy, have garnered significant attention [3]. Among them, TTN gene mutations are of great significance as they are linked to poor prognosis, chemotherapy resistance, and immunosuppression [4]. These mutations are enriched in metastatic TNBC, reflecting increased genomic instability with disease progression [5]. Immune-active TNBC subgroups with TTN mutations often exhibit higher tumor mutational burden (TMB), elevated PD-L1 expression, and reduced tumor immune dysfunction and exclusion (TIDE) scores, suggesting higher responsiveness to immunotherapy [6]. In the TNBC-specific ICAM1-TME study, the high-risk group had an increased TP53/TTN/SYNE1 mutation rate and TMB and was more likely to benefit from immunotherapy [7]. Additionally, the long non-coding RNA TTN-AS1 is upregulated in TNBC and TTN-AS1 knockdown inhibited the proliferation, invasion, and migration of TNBC cells [8]. This finding suggests that the non-coding regulatory axis of the TTN locus exerts a pro-tumor effect in TNBC and possesses potential value for therapeutic intervention.

Furthermore, the presence of TTN mutation and deficiency was found to be correlated with a profound reprogramming of the immune landscape in TNBC [6, 7]. Yet the precise mechanisms by which TTN mutation and deficiency drives MDSC-mediated immune evasion and chemoresistance remain poorly understood.

This study aims to delineate the role of TTN mutation and deficiency in TNBC, focusing on their contributions to chemoresistance and immune suppression. Using whole-exome (WES) and matched RNA sequencing from TNBC patient samples combined with clinical data, we analyze the genomic and transcriptomic profiles linked to TTN mutations and their impact on the tumor microenvironment. Our findings may reveal novel TTN-related therapeutic strategies to improve TNBC treatment outcomes.

## Results

### Multicenter exon nucleotide sequencing was conducted to obtain the target gene TTN

**Fig. 1:**
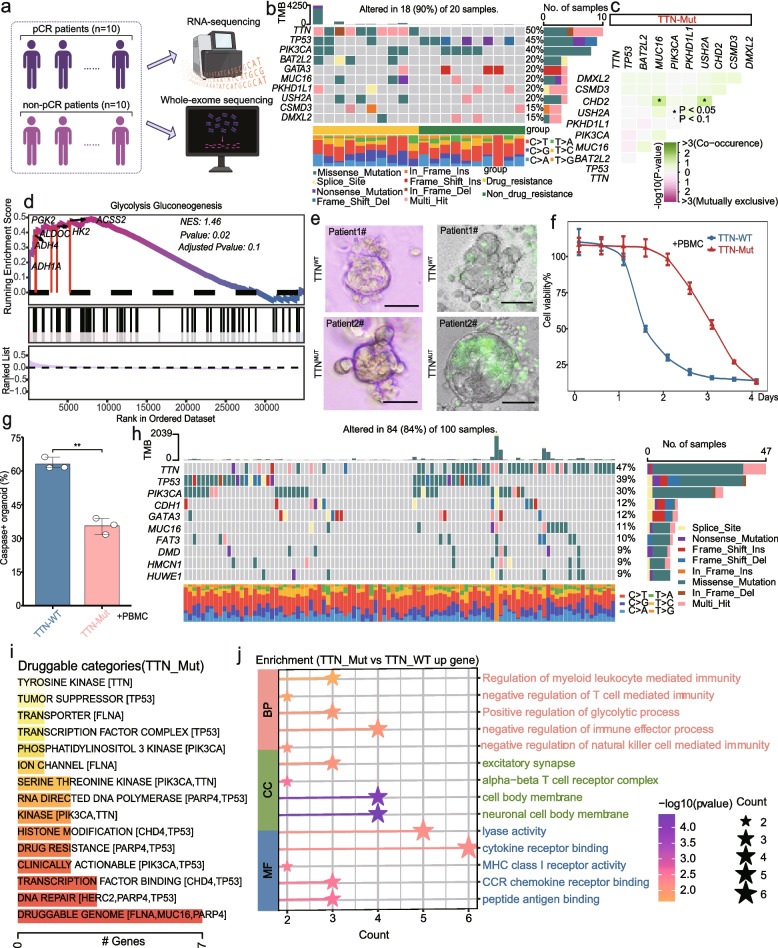
TTN identified as the target gene via multicenter exome sequencing. **a** Experimental design. **b** Single-nucleotide variation in 20 patients. **c** Co-occurring and exclusive mutations in TTN-Mut and TTN-WT. **d** GSEA analysis in TTN-Mut and TTN-WT. **e** Representative images of two TNBC breast cancer organoids treated with Nab-PTX (left) and Caspase 3/7 probe (green) immunofluorescence staining of organoids cocultured with PBMCs (right). Bars, 100 µM. **f** CCK-8 assay detected TTN-Mut and TTN-WT organoid viability. **g** Apoptosis of organoids evaluated using the Caspase3/7 probe. **h** Top 10 mutated genes. **i** Bar plots of drug-gene interactions in TTN-Mut and TTN-WT. **j** GO pathways enriched in TTN-Mut. **P < 0.01

First, we conducted WES and matched RNA sequencing for 20 patients with TNBC in our center, and analyzed the data correlated with the clinical profiles of patients (Fig. 1a). We identified the single-nucleotide variant profile and found the key mutation gene, TTN. There was not much synergy or exclusion between TTN and other mutant targets (Fig. 1b-c). Meanwhile, we observed that the expression level of TTN in TNBC patients and TTN mutations was significantly decreased and enriched in the glycolysis-gluconeogenesis pathway (Fig. 1d, Fig.S1a-b). We constructed corresponding organoids-PBMCs co-culture models of these 20 patients and noticed that organoids with TTN mutation were more likely to resist the Nab-PTX regimen. However, there were no significant differences in resistance between TNBC organoids by TTN mutation alone (Fig. 1e-g, Fig.S2a-b); therefore, we hypothesized that TTN mutation-induced resistance in TNBC relied on the remodeling of its microenvironment**.** We used cohorts collected from different centers and integrated the sequencing data of 100 TNBC patients from authoritative databases, including TCGA and CPTAC (Fig. 1h). The TTN mutation was more likely to trigger various types of synergistic resistance (Fig. 1i). Besides, TTN mutations activated glycolysis while regulating myeloid cell activities (Fig. 1j). We also found that with the presence of PBMCs, deficiency and mutations of TTN showed similar phenotypes of enhanced resistance to Nab-PTX (Fig. 1f-g, Fig.S2c-g). Therefore, we proposed a new concept known as “TTN inactivation”, referring to the TTN gene deficiency and mutation. Through comprehensive evaluation of TNBC patients’ survival data and clinical staging, TTN (FPKM) < 0.07 was defined as TTN gene deficiency. Below this threshold, patients with low TTN expression exhibited poorer survival (Fig.S2h-j). Furthermore, a higher proportion of patients with low TTN expression were classified as Stage III-IV, T3-4, and N1-3 (Fig.S2k-m). TTN patients harboring missense mutations (17%) constitute the largest proportion of and were predominantly classified as N1-3 stages (Fig.S2n-p). Therefore, we identified TNBC samples with TTN (FPKM) < 0.07 or missense mutation as “TTN inactivation”. Meanwhile, IHC scores of "absent" and "weak" were identified as TTN-low expression while "moderate" and "strong" as TTN-high expression (Fig.S2q-r).

### Glycolytic myeloid-derived suppressor cells were enriched in TTN-inactivated triple-negative breast cancer to promote tumor progression

**Fig. 2:**
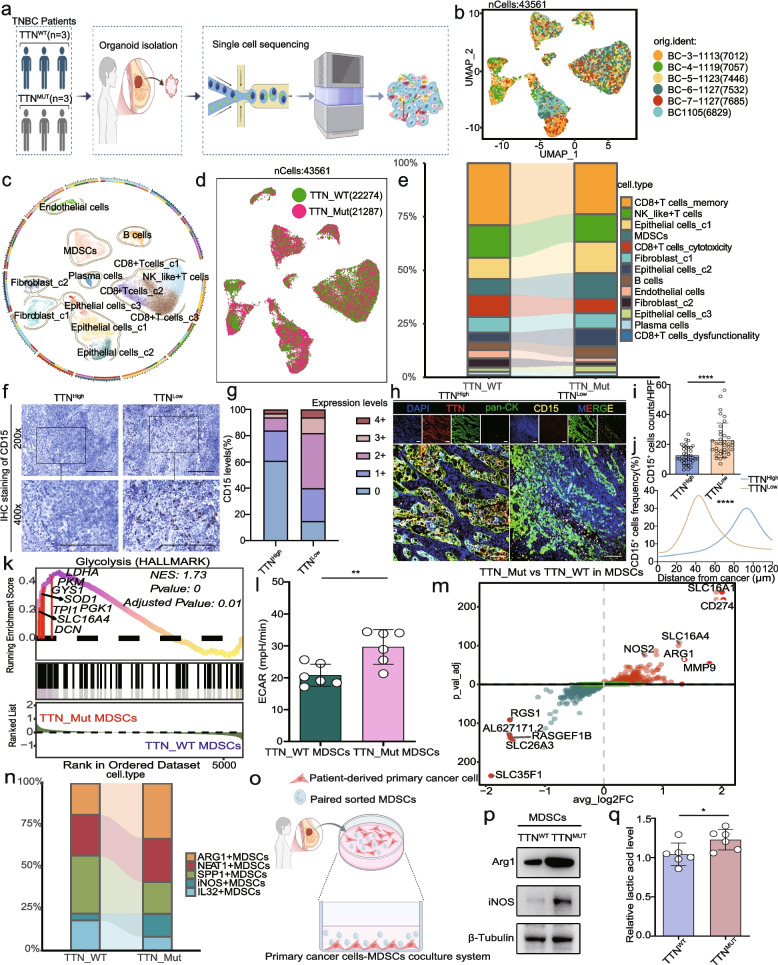
Pro-tumor glycolytic MDSCs were enriched in TNBC with TTN inactivation. **a** Schematic of single-cell sequencing. **b** Sample distribution in scRNA-seq data from six patients. **c** Cell subpopulation distribution via t-SNE. **d** Heatmap of marker gene expression. **e** UMAP visualization of TTN-WT and TTN-Mut groups. **f** Immune microenvironment cell subset changes. **g-h** CD15 expression in patients with TTN-High and TTN-Low by IHC staining. n = 200, bars, 200 µM. **i-k** Multiplexed fluorescent IHC staining of TTN and CD15^+^MDSCs. n = 74, bars, 100 µM. **l** GSEA analysis in MDSCs of TTN-Mut and TTN-WT. **m** The ECAR was compared between MDSCs in TTN-Mut and TTN-WT. **n** Volcano plots of gene expression. **o** MDSC subset changes in TTN-WT and TTN-Mut. **p** Schematic of primary cancer cells and MDSCs coculture. **q** Arg1 and iNOS expression in the MDSCs. **r** Lactic acid levels in the MDSCs. Statistical significance by Student’s t-test. *P < 0.05; **P < 0.01; ****P < 0.0001

Using single-cell transcriptome sequencing, we next investigated the microenvironmental cellular components in TNBC tissues from the TTN-Mut and TTN-WT groups (Fig. 2a-d; Fig.S3), finding that MDSCs was significantly increased in TTN-Mut tumors. Furthermore, a remarkable decrease in the proportion of functional and memory CD8^+^ T cells and a prominent increase in exhausted CD8^+^T cells were observed in TTN-Mut tumors (Fig. 2e). Next, we conducted target gene testing and immunohistochemical staining proving that TTN-Mut tumors enhanced MDSCs infiltration (Fig. 2f-g). By multiplex fluorescent IHC, we found that more MDSCs were distributed in TNBC tissues with TTN mutation, and MDSCs in TTN-Mut tumors were adjacent to tumor cells in terms of spatial localization (Fig. 2h-j). Additionally, we selected TNBC tissue sections with different histopathological types for TTN IHC staining, indicating that TTN status was not associated with tumor pathological type (Fig.S4a-b).

Genomic enrichment analysis (GSEA) indicated that MDSCs in TTN-Mut tumors were enriched in the glycolysis pathway (Fig. 2k). Next, we isolated MDSCs from TTN-WT and TTN-Mut TNBC tumors by flow cytometry. Extracellular acidification rate (ECAR) detection indicated that MDSCs from TTN-Mut tumors showed an enhanced glycolytic phenotype (Fig. 2l). Mitochondrial activity was inhibited in MDSCs from TTN-Mut tumors, suggesting that MDSCs showed an enhanced glycolytic phenotype (Fig.S4c). Additionally, differential expression analysis revealed that MDSCs in TTN-Mut tumors showed higher expression levels of malignant phenotypic markers (Fig. 2m). We also observed an increased proportion of ARG1^+^ and iNOS^+^ MDSCs in TTN-Mut tumors (Fig. 2n). MDSCs obtained from patients’ peripheral blood, co-cultured with primary cancer cells from patients with TTN mutation, secreted more lactic acid and tended to display pro-tumorigenic phenotypes (Fig. 2o-q, Fig.S4d). The similar results could be obtained in mouse cell lines and splenocyte derived MDSCs (Fig.S4e-g).

### DLL4 was essential for TTN inactivation, which enhanced tumorigenesis

**Fig. 3:**
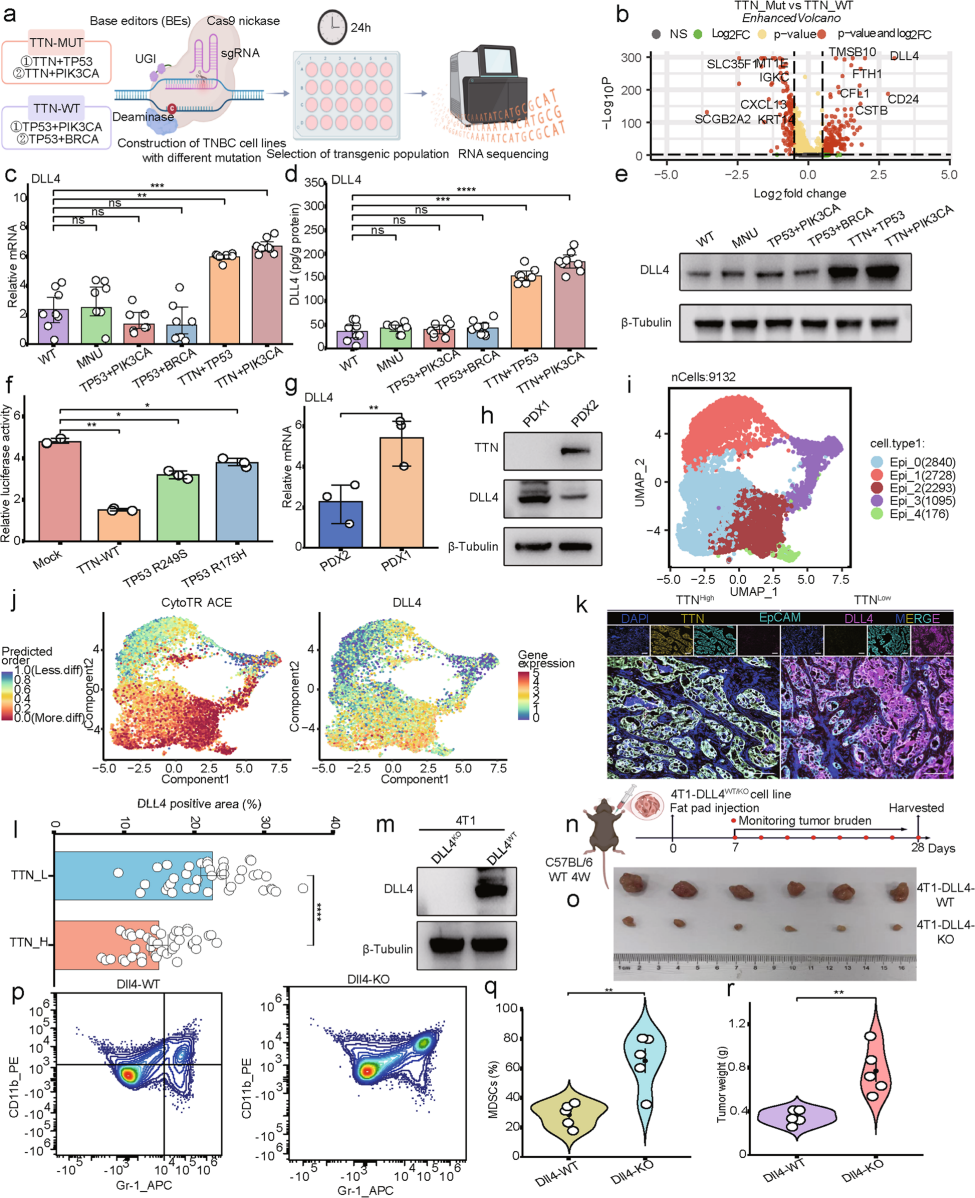
DLL4 was essential for TTN inactivation to promote tumor progression. **a** CRISPR-Cas9 construction of MDA-MB-231. **b** Volcano plot of differentially expressed genes between TTN-WT and TTN-Mut MDA-MB-231 cells. **c** DLL4 mRNA by qPCR. **d** DLL4 content in each group by ELISA. **e** DLL4 protein in each group by Western blotting. **f** Relative luciferase activity of DLL4 in MDA-MB-453 cell lines. **g** DLL4 mRNA in PDX1 and PDX2. **h** DLL4 protein in PDX1 and PDX2. **i** UMAP clustering of tumor epithelial cells. **j** DLL4 expression in tumor subclusters. **k**-**l** DLL4 expression in TTN-High and TTN-Low by IHC staining. n = 80, bars, 100 µM. **m** DLL4 protein in 4T1-DLL4-WT and 4T1-DLL4-KO. **n** Schematic of fat pad injection of 4T1- DLL4-WT/KO cell lines to C57BL/6 mice. **o** Tumor images of 4T1-DLL4-WT and 4T1-DLL4-KO. **p**-**q** MDSC infiltration in 4T1-DLL4-WT and 4T1-DLL4-KO. **r** Tumor weight analyzed in 4T1-DLL4-WT and 4T1-DLL4-KO. **P < 0.01; ***P < 0.001; ****P < 0.0001; ns, no significance

To understand how TTN inactivation regulates the TME, we generated TTN-WT (*Tp53* + *Pik3ca*, *Tp53* + *Brca*) and TTN-Mut (*Ttn* + *Tp53*, *Ttn* + *Pik3ca*) TNBC cell line MDA-MB-231 based on CRISPR-Cas9 gene-editing technology (Fig. 3a). Next, we conducted RNA sequencing of TTN-WT and TTN-Mut tumors and found that DLL4 was the most significantly increased cytokine among the upregulated genes in TTN-Mut tumors (Fig. 3b). q-PCR, ELISA, and Western blotting showed that the expression level of DLL4 significantly increased in TTN-Mut MDA-MB-231 cell lines (Fig. 3c-e, Fig.S5a). Due to the importance of TP53 in TNBC, we measured the regulatory effects of DLL4 mediated by TP53 single-site mutations, observing that TP53 mutation can upregulate the transcriptional activity of DLL4 (Fig. 3f). In addition, we obtained primary cancer cells from TNBC patients who underwent genetic testing. PDX1 cells were obtained from TTN-Mut patients *(Ttn* + *Pten*), and PDX2 cells were obtained from TTN-WT patients (*Ctnnb1* + *Pten*). We found that DLL4 expression was higher in PDX1 cells than in PDX2 cells (Fig. 3g-h, Fig.S5b).

We conducted single-cell RNA sequencing of TTN-Mut (*Ttn* + *Pten*) and TTN-WT (*Ctnnb1* + *Pten*) tumors. After extracting tumor epithelial cells, five clusters were obtained from unsupervised clustering analysis of tumor epithelia (Fig. 3i). CytoTRACE analysis also displayed that DLL4^+^ cells were a subpopulation of malignant tumor cells with relatively high stemness features (Fig. 3j). Multiplex IHC staining showed that DLL4 and EpCAM were co-localized in tumor cells with low expression of TTN (Fig. 3k-l). IHC, ELIAS and Western-blot simultaneously suggested that DLL4 was mainly secreted by TNBC subpopulations with TTN inactivation (Fig.S5c-i).

Subsequently, 4T1-DLL4^WT/KO^ cells were implanted into the fat pads of C57BL/6 mice. Tumor-infiltrated MDSCs and tumor burden were significantly reduced in the DLL4-KO group (Fig. 3m-r, Fig.S5j). These results suggested that DLL4 was essential for TTN inactivation and tumorigenesis.

### TTN inactivation regulated DLL4 expression via the transcription factor NANOS1

**Fig. 4:**
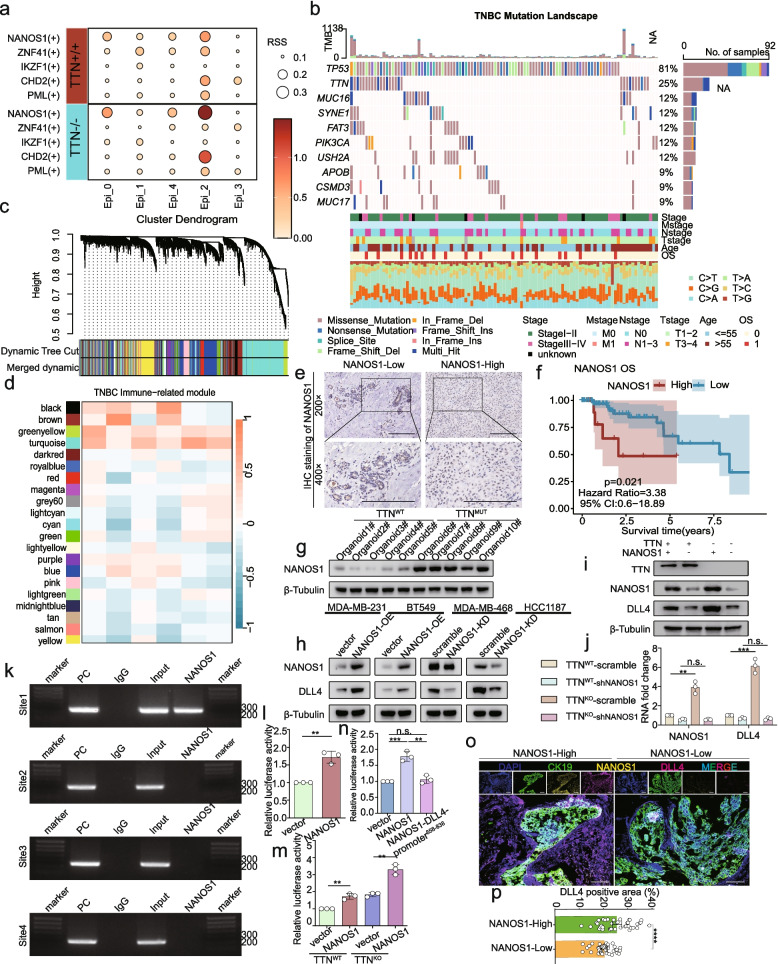
DLL4 expression regulated by TTN inactivation via the transcription factor NANOS1. **a** Transcription factor analysis. **b** Top 10 mutated genes with mutation frequencies and related clinical information in TNBC. **c** Clustering dendrogram of genes. **d** Heatmap of eigengene correlations with immune cells. **e** NANOS1 expression in TTN-High and TTN-Low by IHC staining. Bars, 200 µM. **f** Kaplan–Meier OS for different levels of NANOS1 in TNBC. **g** NANOS1 protein in TTN-WT and TTN-Mut organoids. **h** NANOS1 and DLL4 protein in TNBC cell lines. **i** DLL4 expression in MDA-MB-468 cells line. **j** The mRNA expression levels of DLL4 in TNBC cell lines with varying TTN and NANOS1 expression in MDA-MB-468 cell line detected via qPCR. **k** ChIP assay to validate combination of NANOS1 and DLL4 promoter. **l** Luciferase activity of DLL4 promoter. **m** Luciferase activity in TTN-WT and TTN-KO. **n** Luciferase activity in NANOS1-DLL4-promoter groups. **o**-**p** DLL4 expression in NANOS1-High and NANOS1-Low by IHC staining. n = 80, bars, 100 µM. **P < 0.01; ***P < 0.001; ****P < 0.0001; ns, no significance

Given DLL4 overexpression in TNBC with TTN inactivation, we hypothesized that TTN inactivation may affect DLL4 transcription. pySCENIC transcription factor analysis of single-cell transcriptomes revealed that NANOS1, a transcription factor, was significantly enriched in TTN-deficient cancer cells with high DLL4 expression (Fig. 4a). Mutation data and WGCNA analysis showed that NANOS1 was upregulated in TTN-mutation patients and associated with the immunosuppressive microenvironment (Fig. 4b-d). Subsequent IHC staining revealed that NANOS1 could predict poor survival prognosis (Fig. 4e-f). TNBC organoids also proved that the expression level of NANOS1 was upregulated with TTN-mutation (Fig. 4g, Fig.S6a). Next, we constructed NANOS1 overexpression and knockdown TNBC cell lines (Fig.S6b-c). The expression level of DLL4 was positively correlated with NANOS1 in stable-expression TNBC cell lines, patients paired tumoral and non-tumoral tissues, and mice subcutaneous tumors (Fig. 4h, Fig.S6c-j). And the expression level of DLL4 was significantly suppressed in TTN-deficient cancer cells after NANOS1 knockdown (Fig. 4i-j, Fig.S6k).

We conducted chromatin immunoprecipitation (ChIP) to demonstrate the combination of NANOS1 and the DLL4 promoter (Fig. 4k). Next, we constructed the luciferase reporter plasmid containing the DLL4 promoter (2 kb from the transcription start site) and co-transfected it with the NANOS1 overexpression plasmid into 293 T cells. NANOS1 overexpression enhanced normalized luciferase activity (Fig. 4l). The luciferase activity was further upregulated with deficient TTN (Fig. 4m). Notably, the NANOS1 binding site (RRRCWWGYYY) was located at −858 bp to −838 bp from the DLL4 promoter. Consistently, the regulatory activity of NANOS1 disappeared when the −858 bp to −838 bp region of the DLL4 promoter was deleted (Fig. 4n). However, NANOS1 did not affect the protein stability of DLL4 (Fig.S6l-m) nor promote DLL4 degradation via ubiquitination (Fig.S6n). Co-IP assay indicated that NANOS1 did not directly interact with DLL4 (Fig.S6o-p) nor affect the binding between DLL4 and its E3 ubiquitin ligase MIB1 (Fig.S6q-r). ChIP-qPCR assays confirmed that NANOS1 can directly bind to the promoter region of DLL4 (Fig.S6s). These results indicated that NANOS1 regulated DLL4 expression primarily through transcriptional activation, rather than via post-translational regulation of protein stability. Multiplex IHC staining showed a significant positive co-localization between DLL4 and NANOS1 in TNBC tissues (Fig. 4o-p).

### DLL4 induced MCT4-mediated glycolytic reprogramming and pro-tumorigenic MDSCs in TNBC with TTN inactivation

**Fig. 5:**
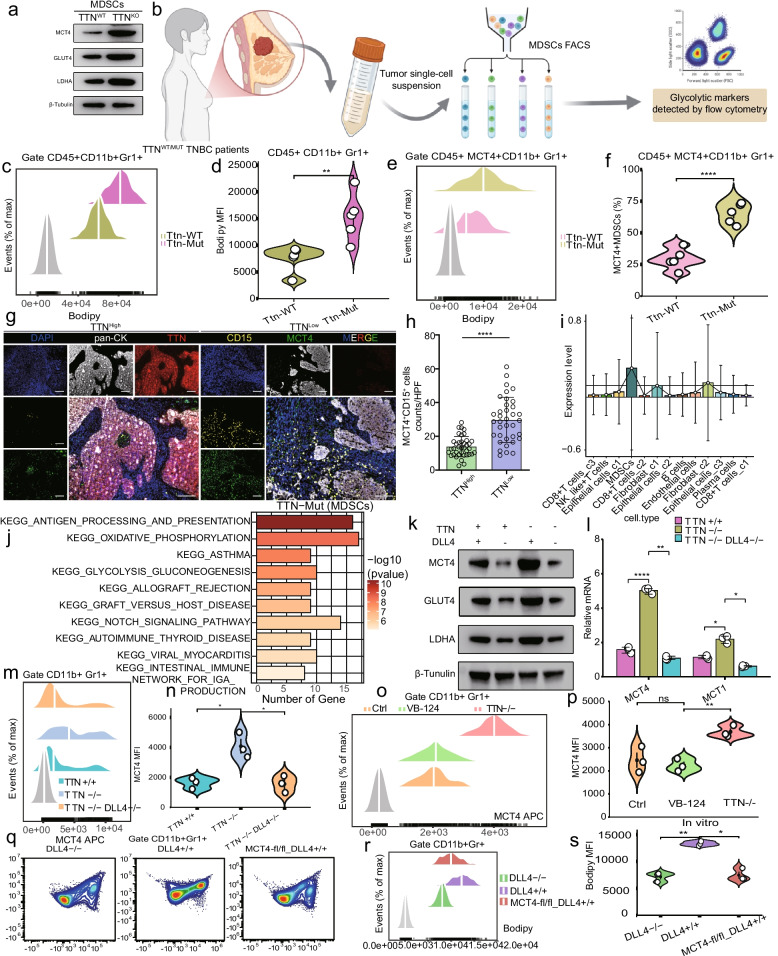
DLL4 participated in metabolic reprogramming of MDSCs mediated by MCT4, promoting tumor progression in TTN inactivation. **a** Glycolytic markers in MDSCs from TTN-WT and TTN-KO. **b** Schematic for marker detection. **c-d** MDSC infiltration in TTN-WT and TTN-Mut. **e–f** MCT4^+^ MDSCs in TTN-WT and TTN-Mut. **g-h** MCT4^+^ CD15^+^ MDSC abundance in TTN-High and TTN-Low by IHC staining. n = 80, bars, 100 µM. **i** Bar plots showing the expression levels of MCT4 in the cell subpopulation. **j** KEGG pathways of MDSCs in TTN-Mut. **k** Glycolytic markers expression in MDSCs cocultured with MDA-MB-468-TTN-WT-scramble, MDA-MB-468-TTN-WT-shDLL4, MDA-MB-468-TTN-KO-scramble, and MDA-MB-468-TTN-KO-shDLL4. **l** The mRNA of glycolytic markers. **m–n** MCT4^+^ MDSC percentage. **o-p** MDSC percentage in Ctrl, VB-124, and TTN-MUT. Statistical significance by Student’s t-test. *P < 0.05; **P < 0.01; ****P < 0.0001; ns, no significance

We next investigated the DLL4-MCT4 axis in MDSCs of TTN-inactivated TNBC. MDSCs cocultured with TTN-deficient cancer cells transfected with a stable TTN-knockout plasmid increased glycolytic markers such MCT4, GLUT4, and LDHA (Fig. 5a, Fig.S7a). Flow cytometry showed that MDSCs from TNBC and TTN mutation patients expressed MCT4 significantly more than MDSCs from TTN-WT patients (Fig. 5b-f). Therefore, we proposed that TTN-inactivated cancer cells promoted MCT4 expression and regulated MDSC glycolysis via secreting DLL4. Multiple IHC stains showed that TTN-mutation TNBC tissues had MDSCs expressing MCT4 (Fig. 5g-h). scRNA-seq from patients’ TNBC tumors with TTN mutation demonstrated MDSCs significantly expressed MCT4. MDSCs enriched NOTCH2 signaling pathway, which was reported positively regulated MCT4 (Fig. 5i-j). Intriguingly, DLL4 knockout in TTN-deficient MDA-MB-231 cells prevented MDSC glycolytic marker upregulation (Fig. 5k-n, Fig.S7b).

We generated DLL4-overexpressing PDX2 (TTN-WT) and DLL4-knockdown PDX1 (TTN-Mut), discovering DLL4 expression unaffected TNBC cell proliferation in vitro (Fig.S7c-d). Therefore, we speculated that DLL4 may influence the immune microenvironment to promote tumorigenesis in TTN-inactivated TNBC. Systematic MDSC depletion by Gr-1 antibody in vivo attenuated tumor growth differences between DLL4-vector and DLL4-OE groups (Fig.S8a-d).

MDSCs cocultured with DLL4-OE TNBC cells increased glycolytic-related enzymes, and vice versa (Fig.S9a-b). We implanted DLL4 overexpression and knockdown PDX cell lines subcutaneously into T/B-deficient nude mice to investigate whether DLL4 affects TNBC development in TTN-deficient mice (Fig.S9c-d), and tumor burden was limited by DLL4 expression in PDX1/PDX2 regardless of TTN genotype (Fig.S9e). In subcutaneous tumors of TTN-MUT and DLL4-OE 4T1 mice, therapy with the MCT4 inhibitor VB-124 decreased tumor-infiltrated MDSCs (Fig. 5o-s). These findings indicated that DLL4 induced MCT4-mediated glycolysis, enhancing MDSC malignancy in TTN-inactivated TNBC.

### DLL4-NOTCH2-MCT4 axis induced MDSCs glycolysis to promote tumorigenesis

**Fig. 6:**
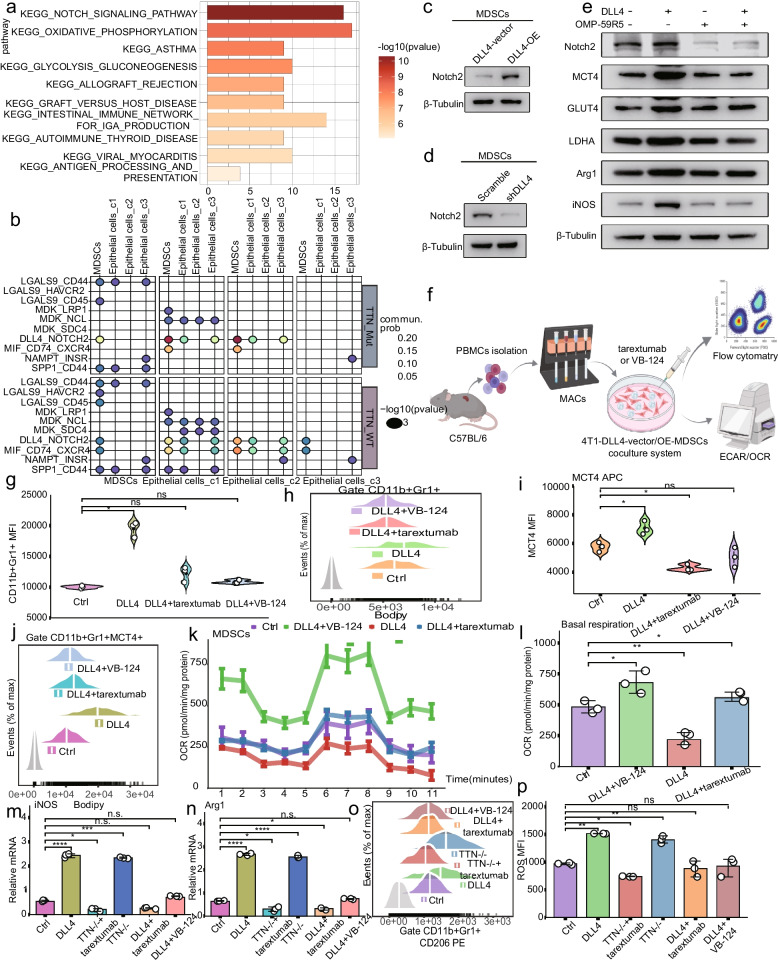
DLL4-NOTCH2-MCT4 axis induced glycolytic reprogramming in MDSCs to form a malignant phenotype. **a** KEGG pathways of DLL4-OE. **b** Ligand-receptor interactions between MDSCs and epithelial cells in TTN-WT and TTN-Mut. **c** Notch2 protein in MDSCs with DLL4-vector and DLL4-OE. **d** Notch2 protein in MDSCs with scramble and shDLL4. **e** Glycolytic and malignant markers expression of MDSCs in DLL4-vector-vehicle, DLL4-OE-vehicle, DLL4-vector-OMP-59R5, and DLL4-vector-OMP-59R5. **f** Experimental design. **g-h** MDSC percentage in different groups by flow cytometry. **i-j** MCT4^+^ MDSCs in different groups. **k-l** OCR analysis of different groups. **m–n** mRNA of iNOS and Arg1. **o** Percentage of CD206^+^ MDSCs in different groups. **p** ROS MFI of MDSCs. Statistical significance by Student’s t-test. *P < 0.05; **P < 0.01; ***P < 0.001; ****P < 0.0001; ns, no significance

The KEGG enrichment pathways showed the NOTCH signaling pathway was remarkably correlated with DLL4 overexpression (Fig. 6a). Meanwhile, scRNA-seq detected DLL4-NOTCH2 (between epithelial cells and MDSCs) receptor-ligand signaling pair in patient-derived TTN-Mut tumors, which is a key pathway for glycolytic-related MCT4 (Fig. 6b). Therefore, we hypothesized that DLL4 may promote MCT4 expression by activating the NOTCH2 signaling pathway. NOTCH2 was significantly increased in MDSCs cocultured with DLL4-OE TNBC cell lines and vice versa (Fig. 6c-d, Fig.S10a-b). We found that RBPJ, a NOTCH signaling pathway transcriptional regulator, promoted MCT4 transcription in MDSCs (Fig.S10c-f), and inhibited by NOTCH2-specific inhibitor OMP-59R5 (Fig.S10g-h). DLL4 exposure didn't upregulate MDSC MCT4 expression after pretreatment with OMP-59R5 (tarextumab) (Fig. 6e, Fig.S10i).

Previous studies revealed DLL4 induced MDSC malignancy via MCT4-mediated metabolic-reprogramming, promoting chemoimmunotherapy resistance. We constructed the TNBC cells-MDSCs coculture system with VB-124/tarextumab to confirm that DLL4 triggered metabolism via the NOTCH2/MCT4 axis (Fig. 6f). Flow cytometry indicated MCT4 and NOTCH2 blockade reduced MDSC infiltration and MCT4 expression (Fig. 6g-j). OCR experiment demonstrated that tarextumab and VB-124 reversed glycolytic activity by blocking DLL4-induced MDSC oxygen consumption (Fig. 6k-l). These finding indicated that DLL4 contributed in MDSC glycolysis via NOTCH/MCT4. Meanwhile, tarextumab and VB-124 inhibited pro-tumorigenic MDSCs (Fig. 6m-p). When MCT4 and NOTCH2 were disrupted, DLL4 couldn’t remodel MDSCs’ malignancy. The “DLL4-NOTCH2-MCT4 axis”formed glycolytic MDSCs in TNBC since NOTCH2 promoted MCT4 expression and both DLL4 and MCT4 were necessary for glycolysis.

### TTN inactivation-derived DLL4 remodeled the anti-tumor immune microenvironment through MCT4^+^ MDSC-CD8^+^ T

It was indicated that MDSCs promoted tumor invasion by suppressing T-mediated anti-tumor immunity. Next, we sorted CD45 + CD3 + tumor-infiltrating T cells from murine subcutaneous 4T1-TTN-WT and 4T1-TTN-MUT tumors for RNA-seq (Fig.S11a). TTN-MUT tumors expressed more T cell-related exhaustion markers (PDCD1, CTLA4, LAG3, and HAVCR2) and infiltrated lower cytotoxic T cells (Fig.S11b-c). Flow cytometry showed TTN-KO TNBC had less number of proliferative CD8 + T than TTN-WT (Fig.S12a). For in vitro T-cell inhibition assays, we separated MDSCs from 4T1-TTN-MUT and 4T1-TTN-WT tumors and cocultured them with splenic T cells (Fig.S11d). MDSCs from TTN-MUT tumors significantly reduced T cell proliferation and IFN-γ production markedly (Fig.S11e-g, Fig.S12b).

We cocultured MDSCs with 4T1-TTN-WT, 4T1-TTN-KO, and 4T1-TTN-KO-DLL4-KO cells, respectively, to investigate the mechanism of MDSCs suppressed T cell-mediated anti-tumor immunity in TTN-inactivated tumors. Next, we generated the MDSC-T cells coculture system (Fig.S11h). MDSCs cocultured with TTN-KO TNBC cells significantly inhibited T cell proliferation and IFN-γ production. However, DLL4 deletion in TTN-KO TNBC abolished these T cell alterations (Fig.S11i-k).

In vivo experiments verified DLL4 downregulation inhibited 4T1-TTN-MUT subcutaneous tumor proliferation and promoted apoptosis (Fig.S11l-o). Thereafter, we implanted 4T1-TTN-KO cells into MDSCs-MCT4^fl/fl^ genetically engineered and wild-type mice’ fat pads, and the tumor burden was decreased after eliminating MCT4^+^MDSCs (Fig.S12c). Additionally, we cocultured MDSCs from MDSCs-MCT4^fl/fl^ and wild-type mice with TTN^WT/MUT^ 4T1 cells. We then cocultured MDSCs with mouse spleen T cells. MCT4 deletion in MDSCs also enhanced T cell proliferation and IFN-γ production (Fig.S12d-e). These findings demonstrated DLL4-MCT4 axis suppressed T cell immunity to support MDSC-mediated immune evasion in TTN-MUT TNBC.

### Glycolytic MDSCs promoted TNBC resistant to Nab-PTX + pembrolizumab

**Fig. 7:**
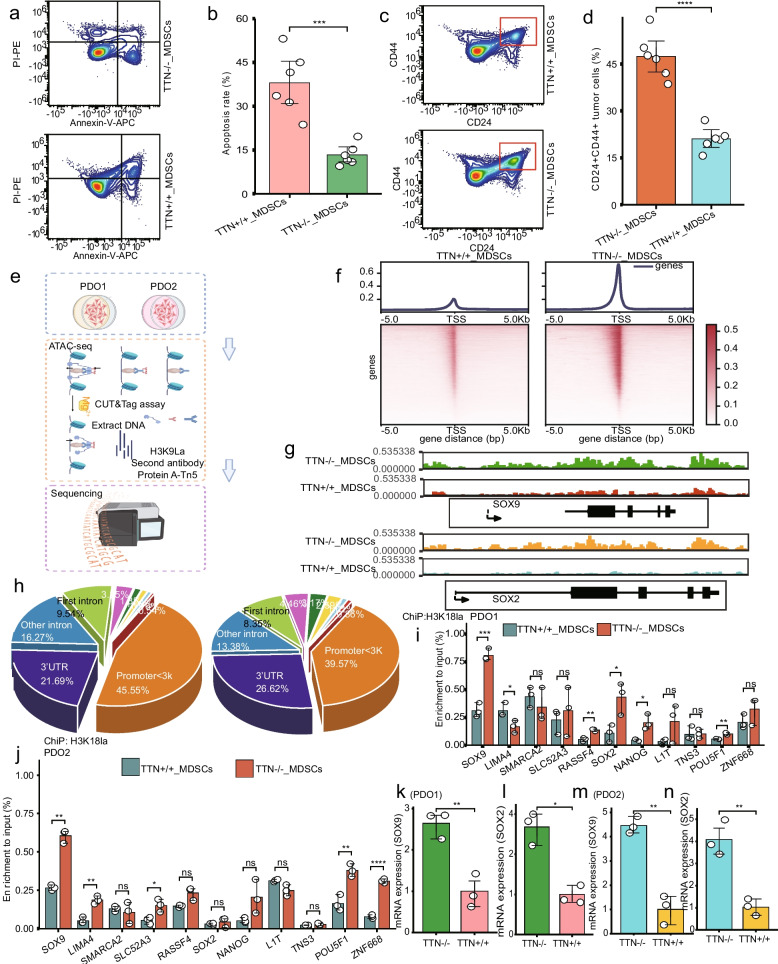
MDSCs mediated by tumoral TTN inactivation enhanced stemness and drug resistance of TNBC cells. **a-b** Percentage of apoptotic TNBC cells cocultured with MDSCs in TTN-WT and TTN-KO by flow cytometry. **c-d** Percentage of CD24^+^CD44^+^ TNBC cells cocultured with MDSCs in TTN-WT and TTN-KO. **e** Schematic for ATAC sequencing. **f** Peak plots of ATAC sequencing of PDO1 and PDO2. **g** ChIP sequencing peak map of SOX9 and SOX2 promoters in PDO1 and PDO2. **h** Chromatin enrichment regions. **i-j** ChIP assay and qPCR validation in PDO1 and PDO2. **k-n** mRNA levels of SOX9 and SOX2 in PDO1 and PDO2. Statistical significance by Student’s t-test. *P < 0.05; **P < 0.01; ***P < 0.001; ****P < 0.0001; ns, no significance

After Nab-PTX + pembrolizumab, MDSCs cocultured with TTN-KO cancer cells attenuated apoptosis and increased CD24 + CD44 + cell subpopulations in TNBC (Fig. 7a-d). Thereafter, we explored how tumoral TTN-MUT-induced MDSCs directly affected TNBC resistant to Nab-PTX + pembrolizumab.

Firstly, MDSCs were cocultured with TTN-WT and TTN-MUT TNBC organoids, respectively. MDSCs then cocultured with TTN-WT organoids, namely PDO1 and PDO2 (from two TTN-WT patients). ATAC-seq of PDO1 and PDO2 indicated that TTN^MUT^_MDSCs group significantly upregulated the chromatin opening state of stemness-associated transcription factor promoter regions including SOX9 and SOX2 (Fig. 7e-h). Therefore, we hypothesized MDSCs might induce Nab-PTX + pembrolizumab resistance by promoting tumor stemness.

It was shown significant glycolytic activity in these MDSCs; therefore, we focused on their direct effect on TNBC histone acetylation. H3K18ac histone acetylation is crucial to cancer cell responsiveness to lactic acid. H3K18la histone ChIP and qPCR experiments confirmed the upregulation of stemness-associated transcription factors (Fig. 7i-j). qPCR showed SOX2 and SOX9 overexpressed in the TTN^MUT^_MDSCs group in both PDO1 and PDO2 (Fig. 7k-n). The TTN^MUT^_MDSCs group of PDO formed more tumor spheres, promoted ALDH1 and tumorigenesis (Fig.S13a-e). P300 is a key enzyme catalyzes histone acetylation modifications, such as H3K18la [9]. Furthermore, p300 and key stemness-related factors were upregulated and p300 was essential for tumor stemness in the TTN-Mut group in both PDO1 and PDO2 (Fig.S13 f-i). These findings suggested TTN-inactivation induced MDSCs enhanced stemness and drug resistance in TNBC.

### Blocking the DLL4-MCT4 axis may sensitize chemotherapy and immunotherapy in TTN-inactivated TNBC.

**Fig. 8:**
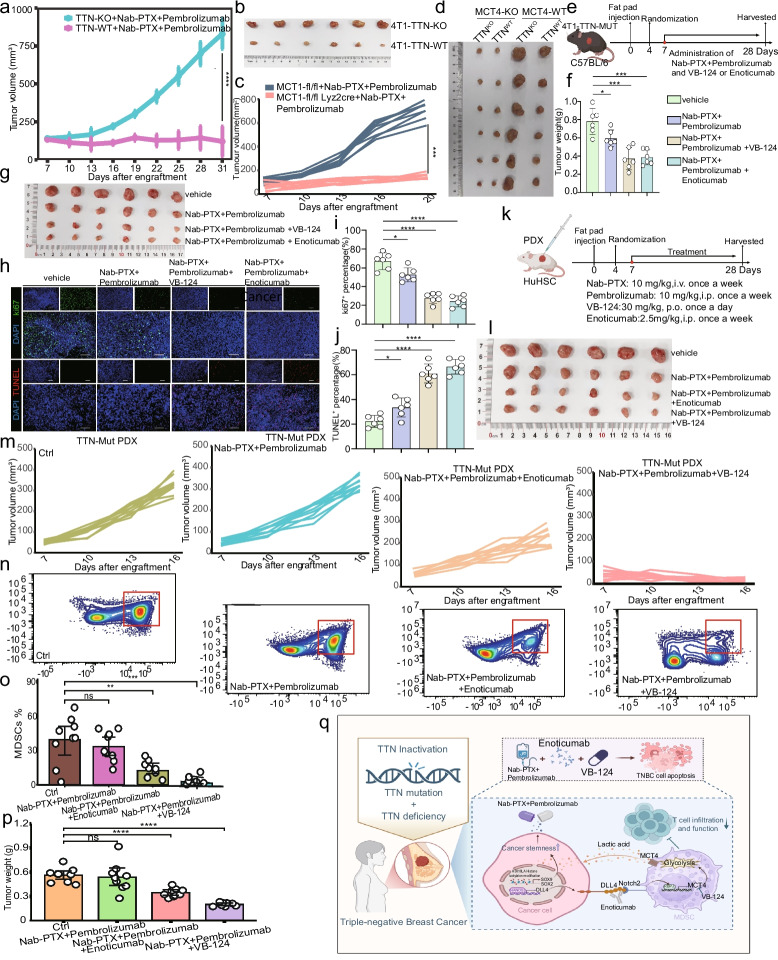
Blocking the DLL4-MCT4 axis as a potential immunotherapy for TNBC with TTN inactivation. **a** Growth curve of 4T1-TTN-WT and 4T1-TTN-KO based on tumor size. **b** Representative tumor images of 4T1-TTN-WT and 4T1-TTN-KO groups. **c** Growth curve of MDSC groups. **d** Representative tumor images of each group. **e** Fat pad injection schematic. **f** Tumor weight of vehicle, Nab-PTX + pembrolizumab, Nab-PTX + pembrolizumab + VB-124, and Nab-PTX + pembrolizumab + enoticumab groups. **g** Representative tumor images of each group. **h-j** Ki67 and TUNEL staining of subcutaneous tumors. **k** PDX subcutaneous injection schematic. **l** Representative tumor images of each group. **m** Tumor growth curves of each group. **n–o** Tumor-infiltrating MDSC percentage in each group. **p** Tumor weight of each group. **q** The schematic illustration of the mechanism. Statistical significance by Student’s t-test. *P < 0.05; **P < 0.01; ***P < 0.001; ****P < 0.0001; ns, no significance

4T1-TTN-WT and 4T1-TTN-KO cells were inoculated into the fat pads of Nab-PTX + pembrolizumab-treated C57BL/6 mice. The 4T1-TTN-KO group exhibited treatment resistance and higher tumor burden (Fig. 8a-b). Given that MDSCs induced by the DLL4-MCT4 axis can promote tumor immune evasion in TNBC with TTN inactivation, we wondered whether blocking the DLL4-MCT4 axis could inhibit tumor growth. Next, 4T1-TTN-WT and 4T1-TTN-KO cells were implanted into the fat pads of MDSCs-MCT4^WT^ and MDSCs-MCT4^fl/fl^ mice, receiving Nab-PTX + pembrolizumab. Both TTN-KO and TTN-WT tumors were sensitive to chemotherapy and immunotherapy in the MDSCs-MCT4^fl/fl^ genetically engineered mouse model (Fig. 8c-d). We subsequently injected 4T1-TTN-MUT cell lines into the fad pads of C57BL/6 mice, and treated them with Nab-PTX + pembrolizumab together with the DLL4 neutralizing antibody enoticumab or the MCT4 inhibitor VB-124 (Fig. 8e). DLL4 or MCT4 blockade significantly limited tumor burden and proliferation compared with Nab-PTX + pembrolizumab alone (Fig. 8f-j). Notably, Nab-PTX + pembrolizumab combined with enoticumab or VB-124 more effectively inhibited tumor growth and significantly decreased the proportion of MDSCs in a humanized mouse model with PDX tumors (Fig. 8k-p). The subcutaneously implanted TTN-KO tumors did not show any response to anti-PD-1 therapy. However, the combination of anti-DLL4 and anti-PD-1 antibodies strongly inhibited tumor growth in TTN-KO TNBC tumors, leading to a complete response in up to 75% of cases (Fig.S14a). Additionally, we investigated whether the co-blockades can induce long-term immunity after re-implantation in complete response mice. Unlike naive mice, the rechallenged mice exhibited tumor rejection after administering anti-PD-1 inhibitors (Fig.S14b). These findings suggest that the DLL4-MCT4 axis is a potential therapeutic target for reducing tumor burden and reshaping the immune microenvironment in TNBC with TTN inactivation.

Moreover, we collected 80 human TNBC specimens and performed base-correlation analysis of DLL4 expression levels based on the treatment response. The proportion of DLL4^+^ tumor cells was significantly higher in TNBC with poor response to neoadjuvant therapy, while the expression level of DLL4 was decreased in TNBC with good response to neoadjuvant therapy (Fig.S15a-b). We next investigated the relationship between the proportion of DLL4^+^ cancer cells in TNBC and the subpopulation of MCT4^+^ MDSCs obtained via reverse convolution of the same single-cell sequencing in the TME. Analysis of the TCGA database showed that the proportion of the DLL4^+^ subpopulation was positively correlated with the abundance of infiltrated MCT4^+^ MDSCs (Fig.S16a). Subsequently, we extracted DLL4^+^ cancer cell subpopulations in single-cell transcriptomes and conducted subpopulation deconvolution mapping to multiple breast cancer datasets, such as TCGA and GEO. A higher proportion of DLL4^+^ subpopulations predicted more metastasis, advanced clinical staging, and higher tumor proliferation index (Fig.S16b-j). In parallel with multiple datasets, we verified that patients with higher expression levels of DLL4 showed shorter relapse-free survival and overall survival (Fig.S16k-o). In conclusion, our findings suggested that the expression level of DLL4 can predict the TNBC development and poor treatment response. Furthermore, blockade of the DLL4 signaling may sensitize tumor cells to chemotherapy and immunotherapy in TNBC with TTN inactivation. TTN, as the biggest known protein, may affect multiple aspects of tumor biology. Thus, we investigated the effects of TTN inactivation on the behavior of TNBC cells and found that TTN inactivation does not affect the migration and metastasis of TNBC cells (Fig.S17a-b).

## Discussion

Recent studies highlight that pathogenic mutations in TNBC correlate with poor prognosis, therapy resistance, and immunosuppression. Specifically, TNBCs harboring pathogenic gene mutations often exhibited poorer prognoses, chemotherapy resistance, and significant immune suppression [10, 11]. This study identifies TTN inactivation as a key alteration linked to synergistic resistance.

Utilizing paired WES and RNA sequencing from 20 TNBC patients, this study identified a distinct mutational profile, with TTN mutations enriched in glycolysis-related pathways. The organoids-PBMCs coculture models revealed that TTN-mutated TNBCs display increased resistance to Nab-PTX, potentially through microenvironmental remodeling. Integration of TCGA and CPTAC data further associated TTN mutation with enhanced MDSC-mediated immunosuppression. The novel concept of "TTN inactivation" is proposed, highlighting TTN-related pathways as a potential therapeutic target in TNBC.

TTN-truncating mutations are strongly associated with homologous recombination deficiency (HRD) [12]. Mechanistically, TTN’s N-terminal domains interact with BRCA1-associated complexes to stabilize replication forks. Confirmed in our TNBC organoid models, loss of TTN function disrupts this process, leading to replication stress and chromosomal instability [13].

Paradoxically, TTN expression levels exhibited context-dependent roles, while TTN was canonically expressed in muscle tissues [14]. High TTN mRNA levels correlate with improved disease-free survival in HER2 + breast cancer, our data revealed low TTN expression in 68% of TNBCs and is associated with enhanced metastasis. Mechanistically, TTN binds EZH2 to inhibit H3K27me3-mediated silencing of metastasis suppressors [15]. Clinically, TTN alterations predict a 34% reduction in pathological complete response to neoadjuvant chemotherapy, positioning TTN status as a predictive biomarker and potential therapeutic vulnerability in TNBC.

The findings indicated that TTN inactivation promotes tumor immune evasion in TNBC by activating the DLL4-NOTCH2-MCT4 signaling axis. TTN inactivation upregulated DLL4, which subsequently activated the NOTCH2 pathway. This activation was essential for the expression of MCT4, a key transporter involved in glycolysis [16, 17]. The enhanced glycolytic activity of MDSCs fosters an immunosuppressive microenvironment and impairs T cell function. Blockade of DLL4 or MCT4 reduced MDSC accumulation and restored T cell activity, highlighting a targetable pathway in TTN-inactivated TNBC (Fig. 8q).

The DLL4-NOTCH2 axis critically regulates tumor progression through vascular and immune modulation. Upon DLL4 binding, NOTCH2 is cleaved by γ-secretase, releasing its intracellular domain (NICD), which translocate to the nucleus and activates targets such as HES1 and MYC [18]. This pathway promotes vascular normalization [19] yet is exploited by tumors to foster abnormal vasculature. TCGA analysis indicates that DLL4 overexpression in TNBC correlates with a 2.3-fold increase in micro-vessel density and elevated metastatic potential [20].

Our study revealed that NOTCH2 activation drives myeloid-derived suppressor cell (MDSC) polarization in TTN-mutant TNBC. The NOTCH2 intracellular domain binds the MCT4 promoter, upregulating this lactate transporter and enhancing glycolytic metabolism in MDSCs. This metabolic shift promotes an acidic, immunosuppressive microenvironment that impairs T cell function [21]. Although DLL4 inhibitors have shown vascular normalization in clinical trials [22], their toxicity supports alternative targeting of downstream effectors such as MCT4. Blocking MCT4 may disrupt this immunosuppressive axis and restore antitumor immunity in TTN-inactivated TNBC.

The limitations of this study were the relatively small sample size, which may have undermined the generalizability of our findings. Additionally, the mechanisms by which TTN inactivation interacts with TME remain to be comprehensively explored, suggesting that future studies should focus on these pathways to bolster therapeutic efficacy for TNBC.

This study demonstrates that TTN inactivation promotes chemoresistance in TNBC by reshaping the tumor immune microenvironment. Genomic and transcriptomic analyses revealed that TTN-inactivated tumors exhibit enhanced glycolysis and increased MDSC infiltration. The DLL4-NOTCH2-MCT4 signaling axis was identified as a key mechanism driving MDSC-mediated immune evasion. These findings highlight potential therapeutic targets and underscore the need for tailored therapies in TNBC with TTN inactivation.

## Materials and methods

### Surgical samples and demographic characteristics of participants

The Cancer Institute of Tianjin Medical University and the Hospital Ethics Committee (bc20240074) approved the use of all samples and participants’ information. All participants provided written consent for the use of their samples and disease information for future studies, following the ethics committee and in accordance with the recognized ethical guidelines of Helsinki.

### Statistical analysis

Visualization was achieved using packages such as ggplot2 (v3.4.2) and pheatmap [23] (v1.0.12). The correlation between the co-occurrence or absence of differentially mutated genes and somatic mutations was evaluated using Fisher's exact test. The relationship between the two variables was examined through either Pearson or Spearman correlation analysis. The significance of between-group differences was determined based on an unpaired Student's t-test (for two-group comparisons) or one-way analysis of variance (ANOVA, for multiple comparisons). Data are expressed as mean ± standard deviation (mean ± SD). Survival analyses were conducted using the Kaplan–Meier approach, with variations in survival examined using the log-rank test. Additionally, multivariate Cox regression analysis was employed to determine the prognostic value of each variable within the framework of risk. All analyses were conducted using GenePattern and R 4.3.0 software.

## Supplementary Information

Supplementary Material 1.

## Data Availability

The datasets used during the current study are available from the public database and the corresponding author upon reasonable request. The expression and mutation data of TCGA was download on October 8, 2024. The single-cell transcriptome of TNBC in our cohort is available from the NODE (National Omics Data Encyclopedia) database (https://www.biosino.org/node/project/detail/OEP00006262).
