# Supplementary material for: Delta-like ligand 4 mediated myeloid-derived suppressor cell metabolic reprogramming promotes neoadjuvant therapy resistance in titin-inactivated triple-negative breast cancer
Source: Mol Biomed. 2025 Dec 2;6:128. doi: 10.1186/s43556-025-00372-6 (PMC12669439; doi:10.1186/s43556-025-00372-6)
Supplement: Supplementary file 1 — Supplementary Material 1. [file 43556_2025_372_MOESM1_ESM.docx]

**DLL4-mediated Myeloid-derived suppressor cell metabolic reprogramming promotes neoadjuvant therapy resistance in TTN-inactivated triple-negative breast cancer**

Yanfang Yang1,2*, Ziyun Liu4*, Parhat Kaysar 4*, Yuxi Han4*, Bo Ni4, Linwei Li1,2, Lina Zhang1,2, Xiaobin Shang5#, Yaoyao Zhou1#, Yongjie Xie4#, Zhansheng Jiang1,3#

Email: [tiyangyf@126.com](mailto:tiyangyf@126.com); liuziyun0115@163.com; [parhat1470611@163.com](mailto:parhat1470611@163.com); [hanyuxi@tmu.edu.cn](mailto:hanyuxi@tmu.edu.cn);nibo0807@163.com; [lilinwei@tjmuch.com](mailto:lilinwei@tjmuch.com); [linazhang2005@126.com](mailto:linazhang2005@126.com); [shangxiaobin626@live.cn](mailto:shangxiaobin626@live.cn); zhouyaoyao@tmu.edu.cn; [2335940013@qq.com;zhjiang@tmu.edu.cn](mailto:2335940013@qq.com;zhjiang@tmu.edu.cn)

1Tianjin Medical University Cancer Institute and Hospital, National Clinical Research Center for Cancer, Key Laboratory of Cancer Prevention and Therapy, Tianjin, Tianjin’s Clinical Research Center for Cancer, Tianjin 300060, China.

2The Second Surgical Department of Breast Cancer, Tianjin Medical University Cancer Institute & Hospital, Tianjin 300060, China.

3Department of Integrative Oncology, Tianjin Medical University Cancer Institute and Hospital, Tianjin 300060, China.

4Pancreas Center, Tianjin Medical University Cancer Institute and Hospital, National Clinical Research Center for Cancer, State Key Laboratory of Drug ability Evaluation and Systematic Translational Medicine, Tianjin Key Laboratory of Digestive Cancer, Tianjin's Clinical Research Center for Cancer, Tianjin, PR China.

5Department of Thoracic Surgery, National Cancer Center/National Clinical Research Center for Cancer/Cancer Hospital, Chinese Academy of Medical Sciences and Peking Union Medical College, Beijing, China.

**These authors shared co-first authorship**: Yanfang Yang, Ziyun Liu, Parhat Kaysar, Yuxi Han

**Corresponding Author**:

Zhansheng Jiang, Department of Integrative Oncology, Tianjin Medical University Cancer Institute and Hospital, National Clinical Research Center for Cancer, Key Laboratory of Cancer Prevention and Therapy, Tianjin, Tianjin’s Clinical Research Center for Cancer, Tianjin 300060, China; E-mail: zhjiang@tmu.edu.cn;

Yongjie Xie, Pancreas Center, Tianjin Medical University Cancer Institute and Hospital, National Clinical Research Center for Cancer, State Key Laboratory of Drug ability Evaluation and Systematic Translational Medicine, Tianjin Key Laboratory of Digestive Cancer, Tianjin's Clinical Research Center for Cancer, Tianjin, PR China; E-mail: 2335940013@qq.com;

Yaoyao Zhou, Tianjin Medical University Cancer Institute and Hospital, National Clinical Research Center for Cancer, Key Laboratory of Cancer Prevention and Therapy, Tianjin, Tianjin’s Clinical Research Center for Cancer, Tianjin 300060, China; E-mail: zhouyaoyao@tmu.edu.cn;

Xiaobin Shang, Department of Thoracic Surgery, National Cancer Center/National Clinical Research Center for Cancer/Cancer Hospital, Chinese Academy of Medical Sciences and Peking Union Medical College, Beijing, China; E-mail: shangxiaobin626@live.cn;

**Supplementary materials and methods**

**Single-cell RNA sequencing and the analysis of tumor heterogeneity**

In this study, TNBC tissue samples (TTN-Mut and TTN-WT), the single-cell sequencing library construction and analysis process is based on the principles of the DNBelab C4 single-cell technology as previously described[1, 2]: (1) The DNBelab C4 technology is a negative-pressure droplet microfluidic-based system. The technology is based on the principle of microdroplet sorting by introducing a proprietary droplet labeling technology (Disc-seq: Droplet-indexed high throughput single-cell sequencing), where labeled capture beads are encapsulated in nanoscaled droplets with single cells or nuclei under negative pressure drive. Each droplet contains individual cells and magnetic beads with unique identifiers, which are gradually converted into DNA nanosphere libraries adapted to the BGI Sequencing Platform (DNBSEQ) through standardized steps such as PCR amplification, restriction endonuclease digestion, and magnetic bead screening in the subsequent library construction process. (2) Sequencing library structure: the original cDNA is transformed into a standardized sequencing data package through multi-stage precision processing - firstly, multiple rounds of PCR amplification are performed to replicate the key gene information, followed by cutting out the invalid fragments with restriction endonuclease, and then extracting the length-qualified DNA fragments through the magnetic bead screening system. Finally, the second amplification forms a standard library adapted to the DNBSEQ sequencing platform, which is then sequenced on the machine. The entire library consists of four core components: CellBarcode: synthetic nucleic acid sequences used to identify cells; UMI: nucleic acid sequences captured from sample tissues; Poly(dT): sequences used for complementary pairing with mRNA poly (A); and Insert: captured RNA fragments. Together, these components form a biological data package with traceability, and each data unit is converted into a digitized signal by the UW sequencer, providing precise input for subsequent analysis.

After generating the gene-cell expression matrix, systematic analysis was performed by Seurat (v4.3.0). Firstly, we constructed an object by “CreateSeuratObject” function, and used “PercentageFeatureSet” to calculate the percentage of mitochondrial genes to complete the data quality control. Subsequently, regularized negative binomial regression normalization was carried out using “SCTransform” function. “FindVariableFeatures” was used to screen for highly variable genes (nfeatures=2000, method="vst"). After dimensionality reduction by principal component analysis (RunPCA, npcs=50), we selected the first 15 principal components based on ElbowPlot and JackStraw significance test, and further constructed the KNN plots using “FindNeighbors”. “FindClusters” function was performed to realize Louvain algorithm clustering. Based on the clustering results, differentially expressed genes were identified by the “FindAllMarkers” function, and manual annotation of cell types was completed by combining markers such as KRT19 (epithelial cells), CD3D/CD3E (T cells), CD14/CD68 (myeloid cells) and COL1A1 (fibroblasts). To eliminate the batch effect between samples, we utilized the “RunHarmony” function of the Harmony (v0.1.1) software package for data batch effect removal, and the final visualized downscaled plots were generated by “RunUMAP”.

“CreateInferCNVObject” was called to construct the analyzed object. CNV was calculated based on the sliding window method, and the aberrant cells of CNV were screened by using the Hidden Markov Model (P<0.01). We took the “CytoTRACE” function of CytoTRACE (v0.3.3)[3] software package to quantify malignant cell stemness, combined with “plotCytoGenes” to visualize the relevant gene expression patterns, and assessed the association between CytoTRACE scores and clinical prognostic markers by Spearman correlation analysis. The key results were visualized multidimensionally by “DimPlot” (UMAP), “FeaturePlot” and “DotPlot” to present a complete picture of the heterogeneous characteristics of TNBC tumors and the trajectory of malignant evolution. The single-cell transcriptome of TNBC in our cohort is available from the NODE (National Omics Data Encyclopedia) database (https://www.biosino.org/node/project/detail/OEP00006262).

**Transcriptome data analysis**

In this study, we integrated the Cancer Genome Atlas (TCGA) triple-negative breast cancer cohort (TCGA-BRCA, ER-/PR-/HER2- subtypes) and the International Cancer Genome Consortium (ICGC) related dataset (BRCA-EU project) to systematically resolve the molecular features of TNBC. RNA sequencing data (FPKM format) and clinical information were obtained from the TCGA GDC Portal (https://portal.gdc.cancer.gov/) and ICGC Data Warehouse (https://dcc.icgc.org/repositories). TCGA expression data is classified as Level 3 and is currently publicly accessible. Upon accessing the official website, navigate to the “Repository” page, filter by cancer type (BRCA represents breast cancer), then select the “Transcriptome Profiling” data type to download the gene expression data. Data standardization (log transformation) was performed by DESeq2 (v1.40.2). We performed differential expressed genes by limma (v3.56.2). Survival analyses were performed using the survival package (v3.5-5).

**Analysis of pySCENIC transcription factors**

In this study, we used the pySCENIC[4] analysis framework based on the Python 3.8 environment to realize the systematic resolution of single-cell transcriptional regulatory networks by integrating multidimensional algorithms. The technical process begins with the construction of a gene co-expression network based on the GRNboost2 algorithm, a random forest regression model that establishes the regulatory relationships between transcription factors and candidate target genes by iteratively calculating the single-cell RNA-seq raw expression matrix. Subsequently, biological validation was performed using the “RcisTarget" tool, and the candidate modules were analyzed for cis-regulatory element enrichment by comparing them to the species-specific DNA motif database (UCSC hg38), and screening for regulatory units that satisfy both the motif similarity scores and co-expression strength criteria. Finally, we quantified the cellular activity characteristics by the “AUCell” algorithm. The AUC scores of regulatory units were calculated based on the target gene expression ordering, and we used the dynamic thresholding method (top 20% expression quartile) to generate binarized activity matrices. The regulatory heterogeneity among cellular subpopulations was visualized by combining with UMAP/TSNE downscaling technology. The output of the analysis results covered the regulatory network topology map, cellular activity heatmap, and the list of differentially regulated modules, which provided a multi-scale chain of evidence for revealing transcription factor-mediated cellular state transitions.

**Pathway enrichment analysis**

In this study, we used a multidimensional gene function annotation strategy, integrating GSEA (Gene Set Enrichment Analysis)[5] and hypergeometric test methods to systematically analyze differential gene function characteristics. Based on the GSEA analysis of predefined functional gene sets (MSigDB v7.5.1), the genes were first sorted according to their association with phenotypes (log2 fold-change sorting matrix), and the distributional characteristics of the gene sets in the phenotypic sorting were evaluated by calculating the normalized enrichment scores (NES), and the false discovery rate was calculated by using the permutation test (1,000 iterations) to determine significant enrichment events. GO and KEGG annotation analyses were also carried out to assess the enrichment of differential genes (FDR ≤ 0.05) in the Gene Ontology database (GO terms) and the KEGG pathway database using the hypergeometric distribution test, with significance thresholds set (p<0.01 and FDR<0.05), and the Benjamini-Hochberg method was used to correction for multiple hypothesis testing. The clusterProfiler[6] software package was used for the whole analysis, and the pathway enrichment status was displayed in various visualization forms, forming a systematic analysis system from the concerted changes of gene sets to the analysis of molecular mechanisms.

**Cell communication analysis**

The cellchat[7] algorithm was used to systematically parse the intercellular communication network and construct a cell-type-specific ligand-receptor interaction model based on single-cell transcriptome data. The normalized expression matrix and cell type annotation information were first integrated to extract the expression profiles specific to each cell subpopulation. Potential ligand-receptor pairs were identified by the “CellChatDB” database, and a two-weight probabilistic model was used to calculate the strength of intercellular communication between cellular subpopulations: the spatial weighting factor was dynamically adjusted based on ligand secretion patterns, and the expression weighting was based on the co-expression level of the ligand-receptor genes (Pearson's correlation coefficient≥0.3). To assess communication network significance, 1000 cell type label substitution tests (Bonferroni corrected p<0.05) were performed to retain biologically significant interactions. Significantly enriched signaling pathways were further identified by hypergeometric tests, and the flow-mediated centrality algorithm was used to quantify key signaling nodes. The analysis results were demonstrated by cellular communication network diagrams, pathway activity heatmaps and ligand-receptor distribution bubble diagrams, and finally, a topological framework of signaling across cellular subpopulations was established.

**WGCNA gene co-expression network**

In this study, the weighted gene co-expression network analysis (WGCNA) framework system was used to parse the transcriptional regulatory network, which was implemented in strict compliance with the standard analysis procedure. Based on the FPKM normalized expression matrix, heteroscedasticity was first eliminated by variance stabilization transformation (voom method), and highly variable genes (top 30% of the coefficient of variation) were screened as the basis for network construction. A soft-threshold power function (power=12, R²＞0.8) was used to construct a scale-free co-expression network, quantify the intensity of co-expression between genes based on the topological overlap matrix (TOM), and apply a dynamic hierarchical clustering algorithm to divide the co-expression modules, set the minimum module size and merge the similar modules by module eigenvectors. The bivariate correlation strategy was used for module-phenotype association analysis to calculate the Pearson correlation coefficients between module eigengenes and target phenotypes, and significant modules were retained after Benjamini-Hochberg correction (FDR < 0.05), and core driver genes were screened based on module membership and gene significance, and all the calculations were performed by the WGCNA software package in R 4.3.0 environment.

**Cell Culture**

The Human TNBC cell lines MDA-MB-231 (RRID: CVCL_0062), MDA-MB-453 (RRID: CVCL_0418), MDA-MB-468 (RRID: CVCL_0419), BT20 (RRID: CVCL_0178), BT549 (RRID: CVCL_1092), HCC1806 (RRID: CVCL_1258), HCC1187 (RRID: CVCL_1247), HCC1937 (RRID: CVCL_0290) and the mouse TNBC cell line 4T1 (RRID: CVCL_0125) were obtained from the Type Culture Collection Committee of the Chinese Academy of Sciences (Shanghai, China). Mycoplasma contamination was excluded in these cell lines at the beginning of this study. STR profiling of all above cell line was conducted and the obtained profiles for our cell lines showed a 100% match with the reference profiles from the ATCC database. The indicated cells were cultured in AdDMEM/F12 (Gibco, Cat#:12634028) or RPMI1640 **(Gibco, Cat#:11875093)** basic medium supplemented with 10% Fetal Bovine Serum (FBS) **(Gibco, Cat#:A5670701)** and 5% penicillin/streptomycin **(Gibco, Cat#:** **15140122)** at 37℃ in a humidified atmosphere of 95% air and 5% CO2. MDSCs were cocultured with 4T1 or CD8^+^ T cells at a ratio of 1:1.

Using the Ficoll-Hypaque density gradient centrifugation method, PBMCs were isolated from the spleen of C57BL/6 mice. PBMCs were maintained in RPMI-1640 supplemented with 10% FBS and 1% Penicillin-Streptomycin. The murine MDSCs and CD8^+^ T cells were respectively sorted from the PBMCs using mice MDSC isolation Kit (Miltenyi Biotec, Cat#:130-094-538) and the mouse CD8^+^ T cell isolation Kit (Miltenyi Biotec, Cat#:130-104-075). T lymphocytes were subjected to polyclonal activation. Briefly, 24-well plates were coated overnight at 4°C with 5 μg/mL anti-mouse CD3ε antibody (Biolegend, Cat#:100339) in sterile PBS. After washing the plates to remove unbound antibody, the isolated T lymphocytes were resuspended in expansion medium [(RPMI-1640 supplemented with 10% FBS, 1% Penicillin-Streptomycin, 5 μg/mL anti-mouse CD28 antibody (Biolegend, Cat#:102115) and 50U/mL mouse IL-2 recombinant protein (Gibco, Cat#:212-12)] at a density of 2 × 10^6^ cells/mL. The cell suspension was then added to the pre-coated plates and cultured at 37°C in a humidified incubator with 5% CO2 for 24h. After incubation, CD8^+^ T cells were collected by centrifugation and cocultured with MDSCs at a ratio of 1:1 for 24 hours at 37°C under 5% CO₂.

We constructed the primary TNBC cancer cells-MDSCs indirect coculture system, where cancer cells were cultured in the lower chamber of the Transwell insert and MDSCs were cultured in the upper chamber of the Transwell insert. Cancer cells were cocultured with MDSCs at a ratio of 1:1 for 24 hours. And after that, we collected the cell suspension in the upper chamber of the Transwell insert (i.e., the cocultured MDSCs). By mouse myeloid-derived suppressor cell isolation kit, MDSCs (CD11b^+^Gr-1^+^) were isolated with high purity from mixed cells to ensure that the lactate detected in subsequent tests came from the MDSCs themselves, rather than residual cancer cells or coculture supernatant. A portion of the separated MDSCs was used to extract proteins for the following Western-blot detection, while the other portion was resuspended in fresh, complete culture medium and inoculated into the 96-well plate. After continuing to culture for 12 hours, the relative lactic acid level detection of MDSCs was performed immediately by the lactate assay kit (Abcam, Cat#: ab65331).

**Bulk RNA sequencing**

Library construction for RNA-seq was performed as described in the TruSeq RNA Sample Preparation Kit. Briefy, isolated total RNA was reverse-transcribed into cDNA with poly-dT primers using the Hifair® kit. Te RNA-seq library was prepared by cDNA synthesis, end repair, 3′ adenylation, adaptor ligation, amplifcation, and product purifcation. Quality control was performed using the Agilent 2100 Bioanalyzer with a DNA chip. After quantifcation with a NanoPhotometer® spectro photometer, libraries were sequenced with paired-end runs on an Illumina NovaSeq 6000 by the CapitalBio.

**Whole-exome sequencing**

The fastp (v0.20.0) was used to filter raw data. The specific conditions were as follows: the adapter in the sequence was identified and cut off in the read, with a minimum length of the reserve being 100 bp. If a read with > 5% “N” bases and/or > 50% low-quality base, the entire pair of reads were removed. Valid sequencing data were aligned to the human reference genome (GRCh38) using the Burrows–Wheeler Aligner (v0.6.1), and the resulting BAM files were preprocessed using the Sentieon (v202010). Sequencing quality statistics were obtained using fastp. The average target sequencing coverage depth of tumor and matched germline samples was approximately 100 ×. To identify all somatic variants in the samples, we used two pipelines (Sentieon TNseq and TNscope) to detect single-nucleotide variants (SNVs) and indels, and matched normal samples were used to exclude germline variations. Somatic mutations were annotated using the ANNOVAR (v20160201)[8]. To obtain an accurate mutation call set, two caller consensus mutations were performed for additional filtering. The bcftools v1.10.2 (https://github. com/samtools/bcftools) was used for further filtering to reduce false positive calls with the following criteria: (1) quality score≥20; (2) FisherStrand≤60.0; (3) StrandOddsRatio≤3; (4) sequencing depth in the region≥30; (5) sequence reads in support of the variant call≥2; and (6) variant allele frequency (VAF)≥0.05. Based on the somatic mutation data, we conducted somatic mutation signature analysis using the decode structSigs1.9.0 R package with the default parameters. The COSMIC signatures were used as the reference to annotate the identified signatures. MuSiC2[9] was used to explore significantly mutated genes (false discovery rate [FDR] < 0.1). Tumor mutation burden was calculated by the Maftools[10] R package. When calculating tumor mutational burden and analyzing mutations related to chemotherapy sensitivity, only mutations with the following functional classifcations were considered: frame_shift_del, frame_shift_ins, in_frame_del, in_frame_ins, missense_mutation, nonsense_mutation, nonstop_mutation, splice_site, and translation_start_site. The processed WES-seq data (level 4) of TCGA can be publicly obtained as MAF format. After accessing the official website, navigate to the “Repository” page, filter by cancer type, and then select the “Simple Nucleotide Variation” data type to download the curated gene mutation data. Somatic copy number alterations (SCNAs) were detected using the CNVkit[11], and genomic regions with significant amplifications or deletions in the samples were summarized by the GISTIC2.0[12]. Tumor purity was estimated by the ABSOLUTE. Germline variants were identified using the Sentieon Haplotyper tool. The ClinVar database[13] was used to annotate known pathogenic and likely pathogenic variants. The 28 cancer predisposition genes were evaluated, which included 12 established breast cancer–predisposition genes (*ATM*, *BARD1*, *BRCA1*, *BRCA2*, *CDH1*, *CHEK2*, *NF1*, *PALB2*, *PTEN*, *RAD51C*, *RAD51D*, and *TP53*) and 16 candidate predisposition genes (*BLM*, *BRIP1*, *CDKN2A*, *ERCC3*, *FANCC*, *FANCM*, *MLH1*, *MRE11A*, *MSH2*, *MSH6*, *NBN*, *RAD50*, *RECQL*, *RINT1*, *SLX4*, and *XRCC2*).

**Construction of triple-negative breast cancer organoids**

The tumor tissues of TNBC patients were minced and digested in the Advanced DMEM/F12 medium **(Gibco, Cat#:12634028)** with collagenase type IV **(Gibco, Cat#:17104019)**, trypsin **(Gibco, Cat#: 25200056)**, gentamicin **(Gibco, Cat#: 15750060)**, and insulin **(Sigma-Aldrich, Cat#: I0516)** at 37°C for 30 min with gentle shaking. Afterwards, the suspension was centrifuged at 500 rcf for five minutes with the supernatant discarded. The pellet was then resuspended in AdDMEM/F12 supplemented with 20U/ml DNase **(StemCell Technologies, Cat#: 07470)** and incubated for five minutes. After centrifugation, the supernatant was discarded, and the pellet was resuspended in AdDMEM/F12. After that, the suspension was strained through a 70μM cell strainer **(Falcon, Cat#: 352350)** and the filtrate was collected and centrifugated to discard the supernatant. Then the pallet was embedded in Corning Matrigel Growth Factor Reduced (GFR) Basement Membrane Matrix **(Corning, Cat#: 356231)** and seeded on the 6-well plate **(Corning, Cat#: 3516)** and cultured in the complete human mammary gland organoid media. The human complete mammary gland organoid media was AdDMEM/F12 media supplemented with 10 μM Y-27632 **(StemCell Technologies, Cat#: 72302)**, 10 mM HEPES **(Gibco, Cat#: 15630080)**, 1× GlutaMAX **(Gibco, Cat#: 35050061)**, 1.25 µg/mL Hydrocortisone **(Sigma-Aldrich, Cat#: H0888)**, 10 ng/mL Heregulin-β1 **(PeproTech, Cat#: 100-03)**, 50 ng/mL hEGF **(PeproTech, Cat#: AF-100-15),** **1×B27 Supplement (Gibco, Cat#: 17504044), 1.25 mM N-Acetylcysteine (Sigma, Cat#: A9165), 10 mM Nicotinamide (Sigma, Cat#: N0636), 500 nM A83-01 (Tocris, Cat#: 2939), 10 nM prostaglandin E2(Cayman Chemical, Cat#: 14010) and 50 ng/mL Human FGF-7 Recombinant Protein** **(Gibco, Cat#: 100-19)[14, 15]**. Organoids were cultured in a humidified atmosphere of 95% air and 5% CO2 at 37°C with the medium changing every 3 days.

Following the successful establishment of patient-derived TNBC organoids and isolation of PBMCs, the co-culture system was established. The co-culture was conducted using the above customized medium consisting of organoid medium components to mitigate anoikis and enhance survival of dissociated organoid cells. PBMCs were maintained in RPMI-1640 supplemented with 10% FBS, 1% Penicillin-Streptomycin, 10 mM HEPES, 1× GlutaMAX and 100 IU/mL recombinant human IL-2 (PeproTech, Cat#:200-02) to support immune cell viability and function[16, 17]. After a minimum of 24 hours of incubation, PBMCs were collected by centrifugation and added directly to the organoid medium. Co-cultures were carried out in ultra-low attachment 96-well U-bottom plates (Corning, Cat#:7007) to minimize adhesion and promote cell-cell contact. Based on preliminary titrations, dissociated organoid cells and pre-activated PBMCs were co-cultured at an effector-to-target (E:T) ratio of 20:1 for 24 hours at 37°C under 5% CO₂[16, 18].

**Organoids apoptosis assay**

For assaying the apoptosis of organoids, we cultured organoids in 50μl Matrigel (Corning, Cat#: 354234) for 3 days. Afterwards, the organoids were isolated with collagenase type IV **(Gibco, Cat#:17104019)**, plated on Matrigel-coated 96-well plates, and treated for 3 days with 1μM Nab-PTX. Then we detected the organoids undergoing apoptosis using a fluorescent caspase 3/7 probe reagent **(Thermo Fisher Scientific, Cat#: C10423)** for 30 min. Apoptotic organoids were monitored by the 520 channel using a BZ-X800 fluorescence microscope.

**Immunohistochemistry**

Using the immunohistochemistry (IHC) kit (Vazyme, Cat#: E-IR-R217), IHC was used to detect TTN, CD15, NANOS1, and DLL4 in tumor tissues. Tumor specimens were fixed in 10% formalin and subsequently embedded in paraffin. Tumor sections were prepared, blocked with 5% BSA for 2 h, and incubated overnight with the primary antibody. They were subsequently incubated for 30 min with the appropriate secondary antibody. Then, the DAB developer was added for further development. Finally, the slides were counterstained with hematoxylin and eosin and examined under a light microscope. IHC score was calculated using staining intensity × percentage of positive cells and was divided into 4 grades: negative (-), weak (+), moderate (++), and strong (+++).

**Multiplex fluorescent immunohistochemistry**

In brief, 5μm TNBC slides were deparaffinized and rehydrated through a graded series of ethanol solutions:(100% 1×10min; 100% 2×10min; 95%×10min; and rinse in 70%) before antigen retrieval in heated Citric Acid Buffer (pH 6.0) in microwave treatment for 15min. Each slide was put through the process of staining by multiplex fluorescent immunohistochemistry kit (Akoya Biosciences, Cat#: NEL871001KT), including a protein block with blocking buffer, followed by primary antibody and corresponding secondary HRP-conjugated polymer. Each HRP-conjugated polymer mediated the covalent binding of a different fluorophore for signal amplification. This reaction was followed by additional antigen retrieval in heated Citric Acid Buffer (pH 6.0) for 15 min to remove bound antibodies. DAPI was used to identify nuclei, and slides were mounted with fluorescence mounting medium. All analyses were conducted with the same type of control. Images were captured by the Zessi fluorescence microscope (400×) and analyzed by ImageJ software.

**Cell Counting Kit-8**

The organoids and PDX cells were seeded in clear, flat-bottom 96-well plates at a density of 1000 cells per well. After cells adherence, organoids and PDX cells were treated with dilution range of Nab-PTX（1μM）or vehicle for 5 days. And then, culture media were replaced with fresh DMEM containing 10% CCK8 (GlpBio, Cat#: GK10039) and plates were incubated for 3 h in an incubator. The absorbance was read at 595nm once a day.

**Mitochondrial Staining with Mito-Tracker Red CMXRos**

TNBC cells were seeded on the 24-well plates and cultured under standard conditions (37°C, 5% CO₂) until 70–80% confluency. Prior to staining, TNBC cells were washed twice with pre-warmed PBS (pH 7.4). Afterwards, cells were incubated with 100 nM Mito-Tracker Red CMXRos (Thermo Fisher Scientific, Cat#: A66443) diluted in serum-free culture medium for 30 min at 37°C in the dark. After removing Mito-Tracker Red CMXRos working solution, TNBC cells were washed three times with PBS. The mitochondria of cells were monitored by the 570channel using BZ-X800 fluorescence microscope.

**Western blotting**

SDS protein lysis buffer supplemented with proteinase inhibitor was used to lyse the cells. Protein concentrations were measured by BCA protein assay. SDS-PAGE electrophoresis separated the proteins in 10% protein gels and transferred them to PVDF membranes. Then the PVDF membranes were blocked in the 5% non-fat dry milk for 1 hours, the PVDF membranes were incubated in primary antibody at 4 ℃ overnight. After three washes with T-BST for 10 minutes, the membranes were incubated at room temperature for two hours with Horseradish peroxidase-conjugated anti-mouse or anti-rabbit secondary antibodies. The blots were detected with a Chemi-Scope exposure machine. The antibodies used in our study were supplemented in **Table S2.**

**Reverse transcription PCR (RT-PCR)**

According to the manufacturer's instructions, the total RNA of cells was extracted using Trizol. The RNA was reverse transcribed to obtain cDNA, and the cDNA levels were analyzed by real-time fluorescence quantitative PCR with 2× SYBR Green qPCR Master Mix. Amounts of mRNA were calculated using the ΔCt method. And each RT-PCR experiment was independently repeated at least three times. GAPDH was used as a loading control. The primers used for RT-PCR in this study were listed in **Table S1**.

**Lactate production assay**

1×10^6^ MDSCs were seeded on the 96-well plates and cultured in conditional culture media. The concentrations of lactic acid in the supernatant were measured by lactate assay kit (Abcam, Cat#: ab65331).

**TTN gene inactivation editing mediated by CRISPR-Cas9-**

Using CRISPR-CasAS9 technology and the online CRISPR design tool (CRISPR.mit.edu), design appropriate sgRNA sequences based on the mutation sites of the TTN gene. Ensure that the sgRNA sequence could be complementary to the target DNA sequence and could guide the Cas9 protein to specific DNA sites. sgRNA was synthesized based on the designed sgRNA sequence using in vitro transcription methods. TNBC cell lines were cultured to an appropriate density. Using Lipofectamine™ 3000, Cas9 protein, and sgRNA were transfected into TNBC cells. The transfected cells were continued to culture to observe their growth and morphological changes, and TTN gene inactivation was detected by qPCR.

**ELISA**

TNBC cell lines (2 × 10^6^ cells) were implanted in 6-well plates and cultured for 72 h, and the conditioned medium was collected after centrifugation at 700 g for 5 min at 4°C. DLL4 protein was quantified using the Human DLL4 DuoSet ELISA (R&D Systems, Cat#: DY1506-05) according to the manufacturer’s instructions. The same culture medium was used as a control.

**Ch-IP assay**

ChIP assays were performed using the ChIP kit **(Thermo Fisher Scientific, Cat#:26157)** according to the manufacturer's instructions. To detect if NANOS1 directly bound to de promoter region of DLL4, TNBC cells were immunoprecipitated with anti-NANOS1 antibody. Then the immunoprecipitated products were detected by PCR and DNA agarose gel electrophoresis.

**Dual-luciferase reporter assay**

TNBC cells were transfected with pCDH-NANOS1 plasmid or control vector (pCDH-vector), which were subsequently transfected with pGL3-DLL4-EBS1-wt and pGL3-DLL4-EBS1-mut, respectively.48 hours later, the supernatants of cell lysates were collected for the luciferase assay. Luciferase activity was determined using the Dual Luciferase Assay System kit as described by the manufacturer’s protocols. Relative luciferase activity of each group was reported as fold induction over the controls.

**Mammary fat pad tumor model in mice**

All protocols in this study were approved by the Committee on the Ethics of Animal Experiments of Tianjin Medical University Cancer Institute and Hospital, in compliance with the Guide for the Care and Use of Laboratory Animals published by the NIH. TNBC cell line 4T1 was resuspended in sterile PBS at 1 × 10⁶ cells/50 μL. Female BALB/c or C57BL/6 mice (6–8 weeks old) were anesthetized with isoflurane (Sigma-Aldrich, Cat#:792632), and a small incision was made in the abdominal skin to expose the fourth mammary fat pad. Cells (50 μL) were injected into the fat pad using a 29-G insulin syringe. For metastasis tracking, luciferase-expressing cells were monitored weekly via bioluminescence imaging (IVIS Spectrum, PerkinElmer) after intraperitoneal D-luciferin injection (150 mg/kg). Tumor volumes were calculated by the following formula: Volume = 1/2 L1 × (L2)2, where L1 is the length of the long axis and L2 is the length of the short axis.

**Flow cytometry (FCM)**

The samples were prepared into a single-cell suspension, and the harvested cells were divided into separate tubes for each antibody staining. Add appropriate concentrations of fluorochrome-conjugated antibodies and incubate for 30 minutes, protected from light. After incubation, cells were washed 3 times. The Beckman fluorescence-activated cell sorter was used to detect the above samples. MDSCs were enriched from single-cell suspensions of mouse spleen or tumor tissues using a negative selection-based mouse MDSC isolation kit **(Miltenyi Biotec, Cat#:130-094-538)**. The gating strategy was employed as follows: ①Debris Exclusion→②Singlet Selection→③Live Cell Gating→④Leukocyte Identification→⑤Total MDSC Gating→⑥Subset Discrimination. For flow cytometric analysis, cells were stained with the following antibody panel: anti-CD45 (clone 30-F11), anti-CD11b (clone M1/70), anti-Ly6G (clone 1A8), anti-Ly6C (clone HK1.4), and a viability dye (e.g., Zombie Aqua™). The data were analyzed using SoftFlow Jo 10.0. The antibodies used in FCM analysis were listed in **Table S2.**

**Extracellular acidification rate (ECAR) and oxygen consumption rate (OCR)**

The Seahorse XF96 Extracellular Flux Analyzer was used to detect cellular ECAR and OCR. On the first day, experimental and control cells were seeded into Seahorse XF96 cell culture microplates, and the XFe96 sensor cartridges were hydrated. Each group should undergo at least 5 repeated measurements. The next day, in order to perform ECAR testing, the microplate was incubated with basic culture medium (containing 1 mM L-glutamine, without glucose) for 1 hour before the measurement. ECAR was measured by continuous injection of glucose, oligomycin and 2-deoxyglucose (final concentration: 10 mM, 1 μM and 50 mM respectively). To perform OCR detection, microplates were incubated with basic culture medium (17 mM glucose, 1 mM sodium pyruvate, 2 mM L-glutamine, pH7.4) for 1 h before measurement. OCR was measured by continuous injection of Oligomycin, FCCP and Rotenone/Antimycin (final concentration: 1, 1, and 0.5 µM, respectively).

**ATAC-Seq analysis**

Open chromatin regions were profiled using the ATAC-seq protocol. Briefly, 50,000 viable cells were lysed, and nuclei were tagged with Tn5 transposase at 37°C for 30 min. DNA was purified, amplified with Nextera primers (5–10 cycles), and size-selected (100–700 bp) using AMPure XP beads. Libraries were validated on a Bioanalyzer and sequenced on an Illumina NovaSeq 6000 (PE150, 50M reads/sample). Reads were aligned to mm10/hg38 (Bowtie2), peaks called with MACS2 (q < 0.05), and differential accessibility analyzed via DESeq2 (FDR < 0.1). Data are normalized to sequencing depth and presented as mean ± SD (**P < 0.01).

**Sphere Formation Assay**

Single-cell suspensions were prepared from breast cancer organoids and seeded into ultra-low attachment 6-well plates at a density of 1,000 cells per well in serum-free breast cancer organoid medium. The plates were cultured for 7 days in a humidified incubator at 37°C with 5% CO₂, in conditions that preserve the important features of the original tumor, such as cellular heterogeneity and self-renewal capacity. After 7 days, the number of spheres with a diameter larger than 75 μm was counted using an inverted microscope (Keyence BZ-X800). Sphere formation efficiency was calculated as the ratio of the number of spheres to the total number of seeded cells.

**ALDEFLUOR Assay**

Single-cell suspensions were prepared from breast cancer organoids by dissociating them with collagenase IV at 37°C for 30 minutes. The resulting single-cell suspension was filtered through a 70 μm mesh. The cells were then incubated with ALDEFLUOR reagent **(Stemcell, Cat#:** **01700)** according to the manufacturer's instructions. ALDEFLUOR is a fluorescent substrate that is metabolized by aldehyde dehydrogenase (ALDH) to produce a bright fluorescent signal. To ensure specificity, the ALDH activity was inhibited in a negative control group by pre-incubating the cells with 1 mM Diethylamino benzaldehyde **(Stemcell, Cat#:** **01701)**, an ALDH inhibitor.The cells were analyzed by flow cytometry.

**In vivo limited dilution assay**

The BALB/c mice were randomized into different groups and PDOs with different dilutions were subcutaneously injected in the fat pad of BALB/c mice. Then the sizes of the tumor were monitored every three days. The stem cell frequency was calculated by the website http://bioinf.wehi.edu.au/software/elda/.

**Co-IP**

Cell lysates were prepared by lysis buffer (150 mM NaCl, 10 mM HEPES, pH 7.4, 1% NP-40) containing complete protease inhibitor tablet and cocktail protein inhibitor. Regarding endogenous immunoprecipitation, cell lysates were subjected to immunoprecipitation with specific antibody or control IgG, followed by the immunoblotting with the indicated antibodies. Besides, immunoprecipitation was simultaneously subjected to a reverse experiment.

**References**

1. Han L, Wei X, Liu C, Volpe G, Zhuang Z, Zou X et al. Cell transcriptomic atlas of the non-human primate Macaca fascicularis. Nature. 2022;604(7907):723-31. doi:10.1038/s41586-022-04587-3.

2. Lei Y, Cheng M, Li Z, Zhuang Z, Wu L, Sun Y et al. Spatially resolved gene regulatory and disease-related vulnerability map of the adult Macaque cortex. Nat Commun. 2022;13(1):6747. doi:10.1038/s41467-022-34413-3.

3. Gulati GS, Sikandar SS, Wesche DJ, Manjunath A, Bharadwaj A, Berger MJ et al. Single-cell transcriptional diversity is a hallmark of developmental potential. Science. 2020;367(6476):405-11. doi:10.1126/science.aax0249.

4. Van de Sande B, Flerin C, Davie K, De Waegeneer M, Hulselmans G, Aibar S et al. A scalable SCENIC workflow for single-cell gene regulatory network analysis. Nat Protoc. 2020;15(7):2247-76. doi:10.1038/s41596-020-0336-2.

5. Subramanian A, Tamayo P, Mootha VK, Mukherjee S, Ebert BL, Gillette MA et al. Gene set enrichment analysis: a knowledge-based approach for interpreting genome-wide expression profiles. Proceedings of the National Academy of Sciences of the United States of America. 2005;102(43):15545-50. doi:10.1073/pnas.0506580102.

6. Xu S, Hu E, Cai Y, Xie Z, Luo X, Zhan L et al. Using clusterProfiler to characterize multiomics data. Nat Protoc. 2024;19(11):3292-320. doi:10.1038/s41596-024-01020-z.

7. Jin S, Plikus MV, Nie Q. CellChat for systematic analysis of cell-cell communication from single-cell transcriptomics. Nat Protoc. 2025;20(1):180-219. doi:10.1038/s41596-024-01045-4.

8. Wang K, Li M, Hakonarson H. ANNOVAR: functional annotation of genetic variants from high-throughput sequencing data. Nucleic acids research. 2010;38(16):e164. doi:10.1093/nar/gkq603.

9. Fan J, Lyu Y, Zhang Q, Wang X, Li M, Xiao R. MuSiC2: cell-type deconvolution for multi-condition bulk RNA-seq data. Brief Bioinform. 2022;23(6). doi:10.1093/bib/bbac430.

10. Mayakonda A, Lin DC, Assenov Y, Plass C, Koeffler HP. Maftools: efficient and comprehensive analysis of somatic variants in cancer. Genome Res. 2018;28(11):1747-56. doi:10.1101/gr.239244.118.

11. Talevich E, Shain AH, Botton T, Bastian BC. CNVkit: Genome-Wide Copy Number Detection and Visualization from Targeted DNA Sequencing. PLoS Comput Biol. 2016;12(4):e1004873. doi:10.1371/journal.pcbi.1004873.

12. Mermel CH, Schumacher SE, Hill B, Meyerson ML, Beroukhim R, Getz G. GISTIC2.0 facilitates sensitive and confident localization of the targets of focal somatic copy-number alteration in human cancers. Genome biology. 2011;12(4):R41. doi:10.1186/gb-2011-12-4-r41.

13. Toledo RA. Inflated pathogenic variant profiles in the ClinVar database. Nat Rev Endocrinol. 2018;14(7):387-9. doi:10.1038/s41574-018-0034-0.

14. Sachs N, de Ligt J, Kopper O, Gogola E, Bounova G, Weeber F et al. A Living Biobank of Breast Cancer Organoids Captures Disease Heterogeneity. Cell. 2018;172(1-2):373-86.e10. doi:10.1016/j.cell.2017.11.010.

15. Dekkers JF, van Vliet EJ, Sachs N, Rosenbluth JM, Kopper O, Rebel HG et al. Long-term culture, genetic manipulation and xenotransplantation of human normal and breast cancer organoids. Nat Protoc. 2021;16(4):1936-65. doi:10.1038/s41596-020-00474-1.

16. Dijkstra KK, Cattaneo CM, Weeber F, Chalabi M, van de Haar J, Fanchi LF et al. Generation of Tumor-Reactive T Cells by Co-culture of Peripheral Blood Lymphocytes and Tumor Organoids. Cell. 2018;174(6):1586-98.e12. doi:10.1016/j.cell.2018.07.009.

17. Cattaneo CM, Dijkstra KK, Fanchi LF, Kelderman S, Kaing S, van Rooij N et al. Tumor organoid-T-cell coculture systems. Nat Protoc. 2020;15(1):15-39. doi:10.1038/s41596-019-0232-9.

18. Li K, Liu C, Sui X, Li C, Zhang T, Zhao T et al. An organoid co-culture model for probing systemic anti-tumor immunity in lung cancer. Cell Stem Cell. 2025;32(8):1218-34.e7. doi:10.1016/j.stem.2025.05.011.

**Supplementary Figures and Legends**


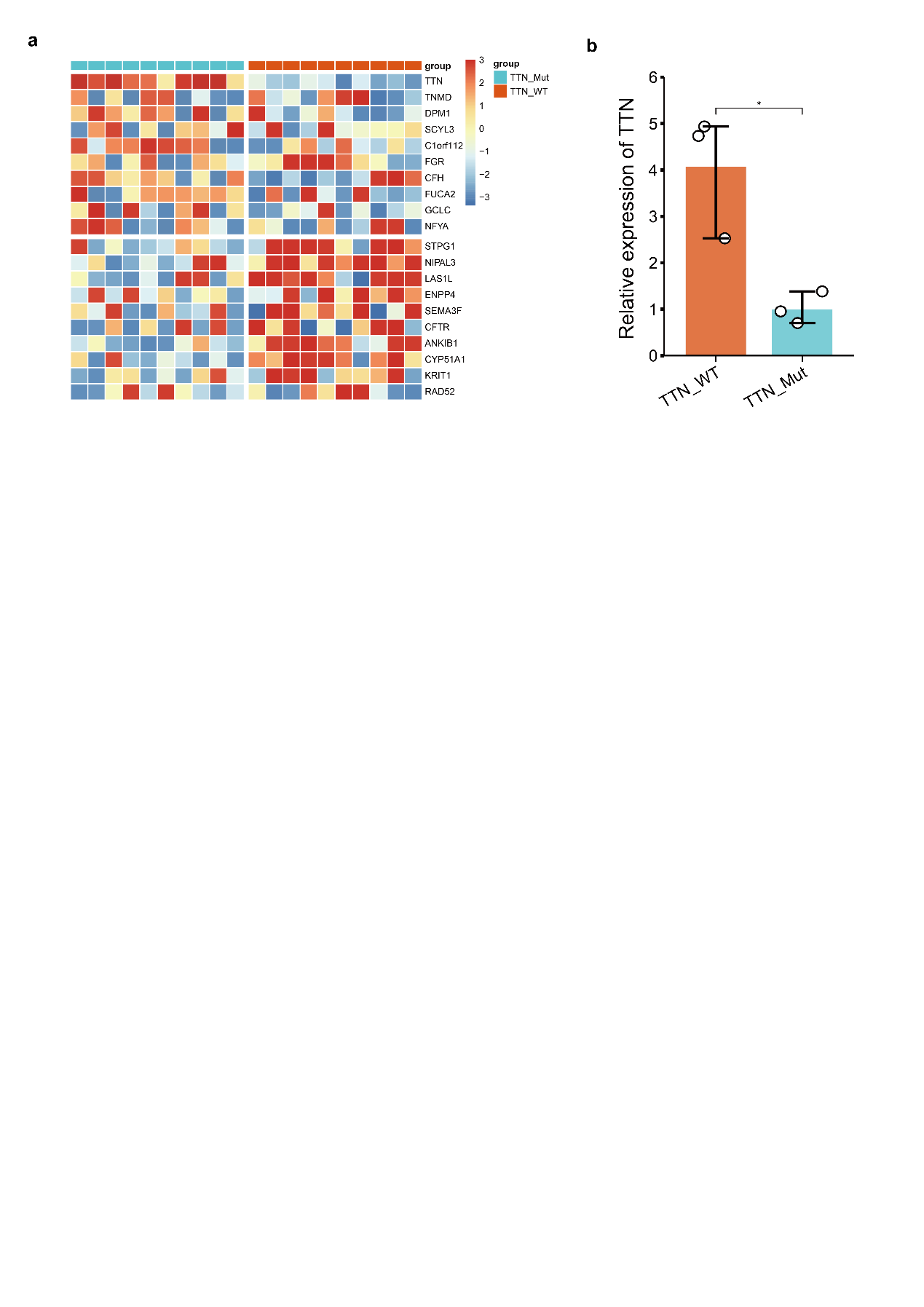


**Fig.S1. TTN enrichment pathway and expression level in triple-negative breast cancer patients with TTN-mutation.**

**(a)** The heatmap displayed the genes related to glycolysis and gluconeogenesis pathways along with correlation coefficients. **(b)** The expression level of TTN of TTN-WT and TTN-Mut PDX by qPCR. Unpaired Student’s t-test was used for statistical analysis. *P<0.05.

**
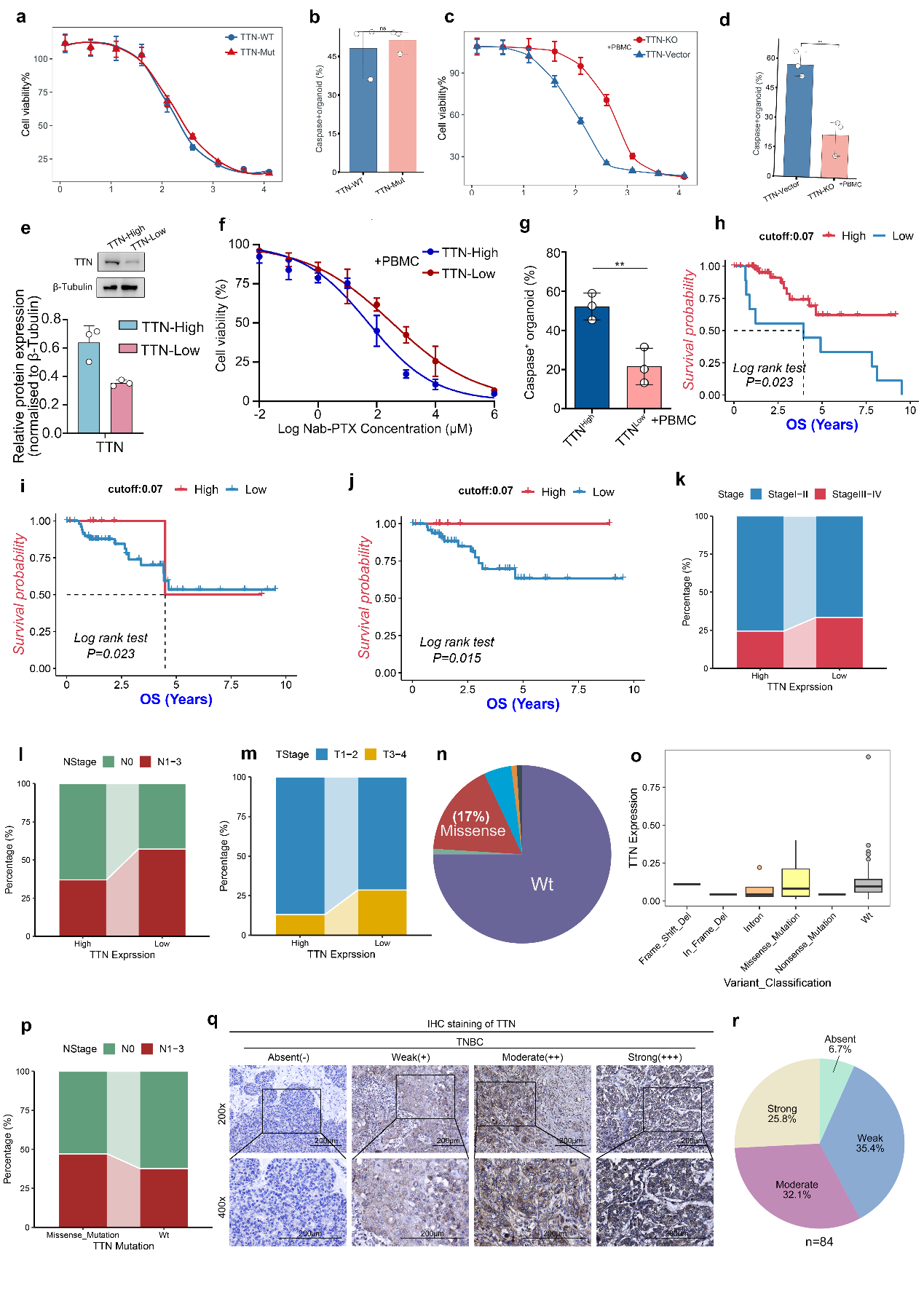
**

**Fig.S2. The cell viability and apoptotic levels of related triple-negative breast cancer organoids.**

**(a)** The cell viability curve of TTN-Mut and TTN-WT organoids, which were not cocultured with PBMCs, was detected by CCK-8 assay. **(b)** The apoptotic levels of TTN-Mut and TTN-WT organoids, which were not cocultured with PBMCs, were evaluated by Caspase3/7 probe. **(c)** The cell viability curve of TTN-vector and TTN-KO organoids, which were cocultured with PBMCs, was detected by CCK-8 assay. **(d)** The apoptotic levels of TTN-vector and TTN-KO organoids, which were cocultured with PBMCs, were evaluated by Caspase3/7 probe. **(e)** The protein expression levels of TTN in the TTN-High and TTN-Low PDOs were detected by Western blot. **(f)** The cell viability curve of TTN-High and TTN-Low organoids, which were cocultured with PBMCs, was detected by CCK-8 assay. **(g)** The apoptotic levels of TTN-High and TTN-Low organoids, which were cocultured with PBMCs, were evaluated by Caspase3/7 probe. **(h-j)** Survival curve comparing high expression and low expression of TTN, under the cutoff of 0.07 FPKM, overall survival **(h)**; progress-free survival **(i)**; disease-specific survival **(j)**. **(k-m)** Bar plot showing the different clinical features percentage in high and low-expressed TTN groups. **(n)** Pie chart describing the percentage of various TTN variant classifications. **(o)** Box-plot showing TTN expression level in different TTN variant classifications. **(p)** The percentage of N stage in TTN-missense mutation and TTN-WT group. **(q)** Representative IHC staining of TNBC tissues with different TTN expressions. **(r)** Distribution of TTN IHC results in 84 TNBC tissues. Unpaired Student’s t-test was used for statistical analysis. *P<0.05; **P<0.01; ns, no significance.

**
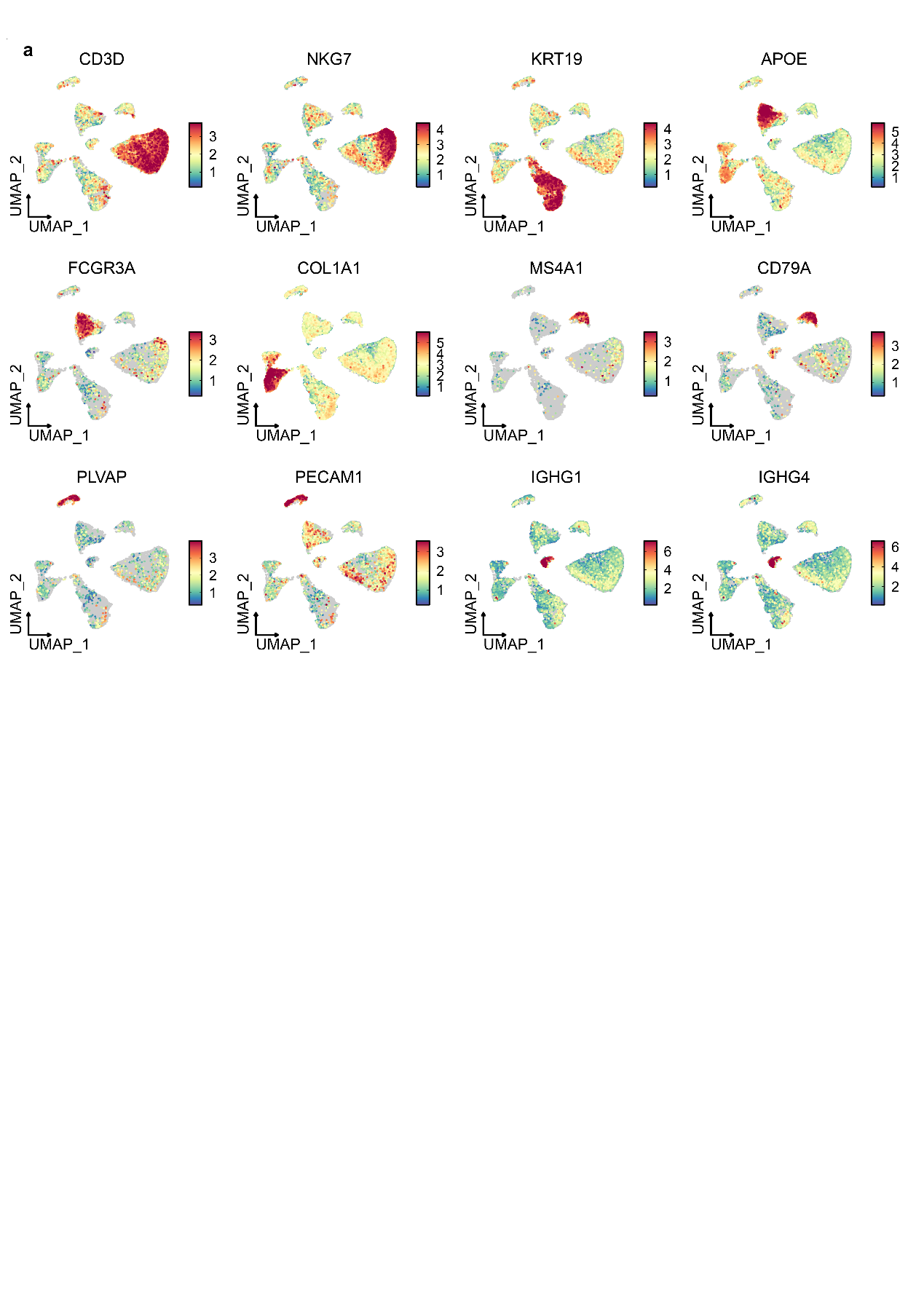
**

**Fig.S3. Identification of different cell types using various cell markers and UMAP dimensionality**


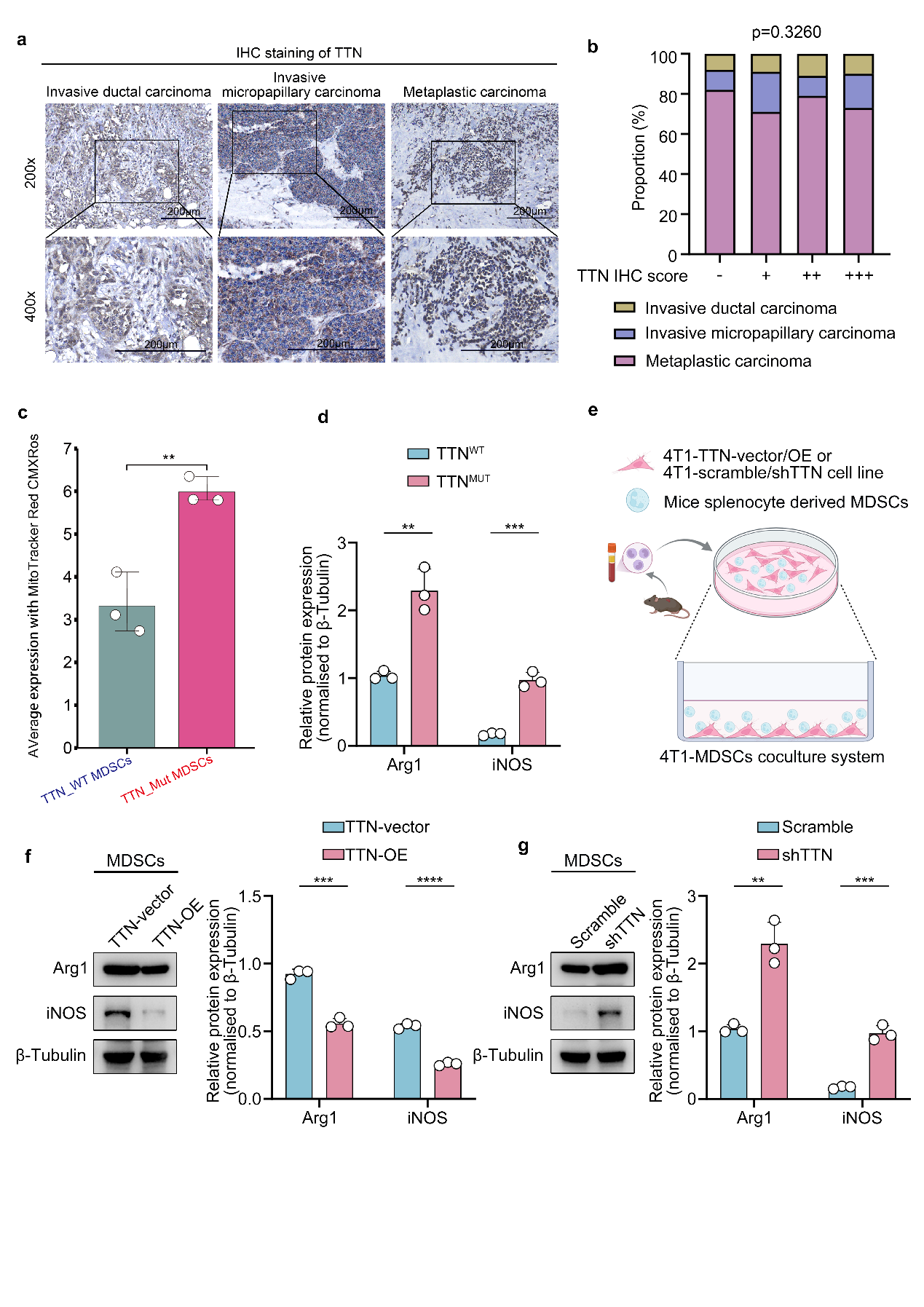


**Fig.S4. The proportion of myeloid-derived suppressor cells with malignant phenotypes increased in TTN inactivation tumors.**

**(a)** Representative IHC staining of TTN in TNBC tissues with different histological and pathological types. **(b)** The proportional distribution of TTN IHC score in different TNBC histopathological types. **(c)** The percentage (%) fluorescence intensity of Mitotracker Red CMX-Ros/DAPI staining in the MDSCs from patients’ derived TTN-WT and TTN-Mut TNBC tumors. **(d)** The statistical analysis of protein levels in Fig.2q. **(e)** Schematic illustration of the 4T1 cell lines and mouse-derived MDSCs coculture system. **(f)** The expression level of Arg1 and iNOS in the MDSCs of TTN-vector and TTN-OE groups was determined by Western blot. **(g)** The expression level of Arg1 and iNOS in the MDSCs of TTN-scramble and shTTN groups by Western blot. **P<0.01; ***P<0.001; ****P<0.0001.

**
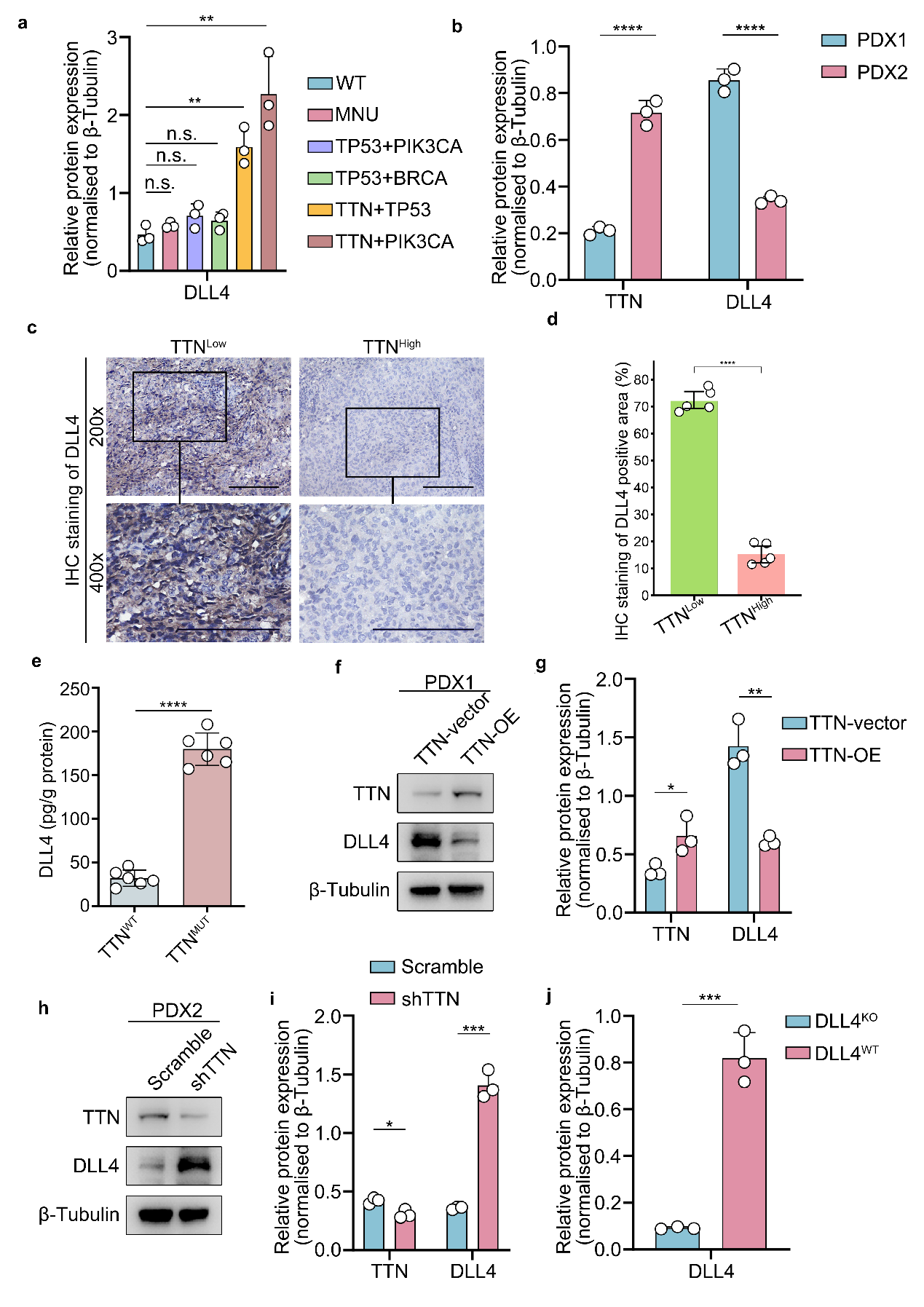
**

**Fig.S5. DLL4 is crucial for TTN inactivation to drive tumor growth.**

**(a)** The statistical analysis of protein levels in Fig.3e. **(b)** The statistical analysis of protein levels in Fig.3H. **(c-d)** The DLL4 expression levels in TTN-High and TTN-Low patients were detected by IHC staining. bars, 200 µM. **(e)** The DLL4 protein contents in TTN-WT and TTN-MUT tumor supernatant of TNBC patients were detected by ELISA. **(f-g)** The protein expression levels of TTN and DLL4 in PDX1-TTN-vector and PDX1-TTN-OE were detected by Western blot. **(h-i)** The protein expression levels of TTN and DLL4 in PDX2-TTN-scramble and PDX2-shTTN were detected by Western blot. **(j)** The statistical analysis of protein levels in Fig.3M. *P<0.05; **P<0.01; ***P<0.001; ****P<0.0001.

**
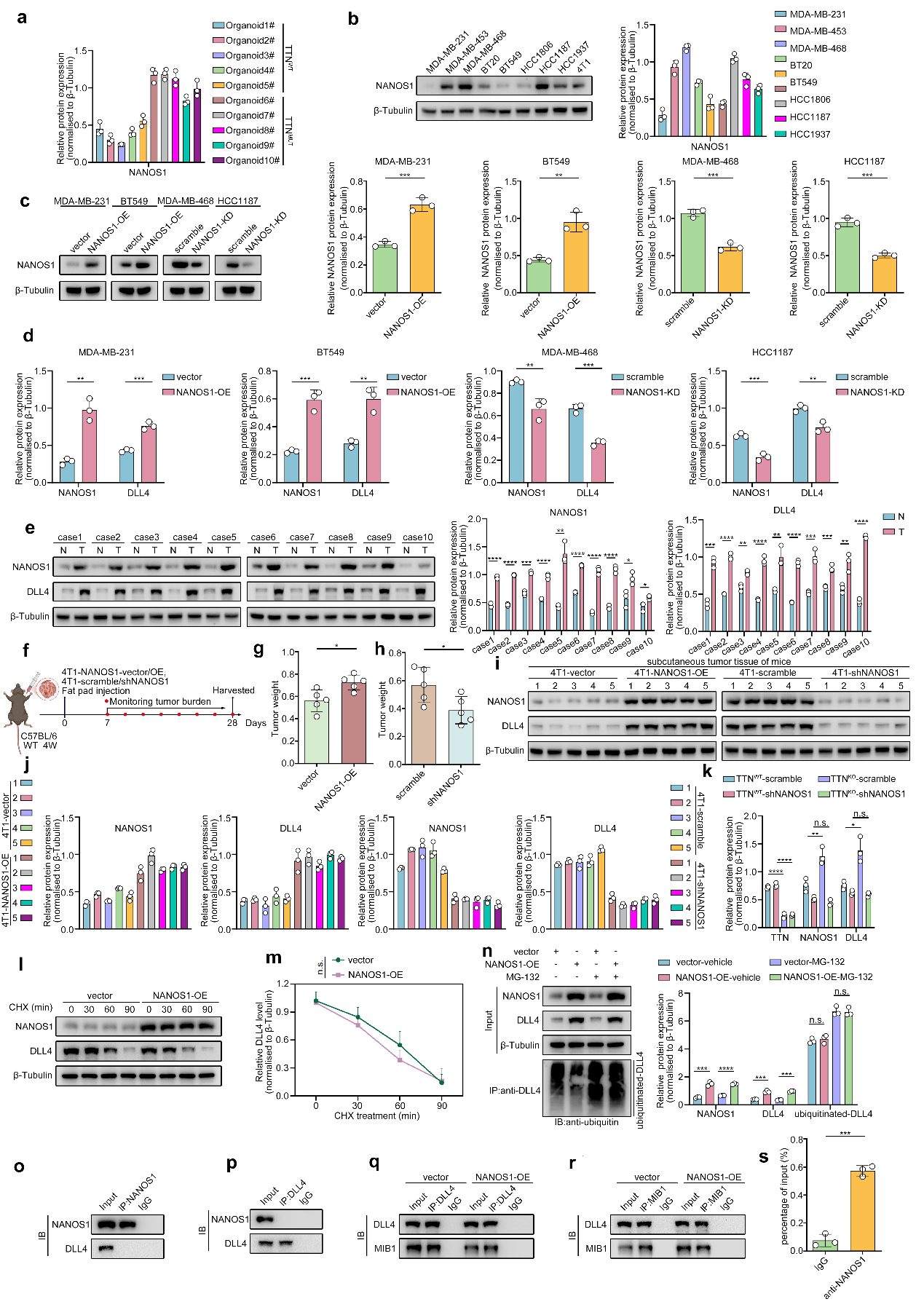
**

**Fig.S6. Tumoral NANOS1 transcriptionally upregulated the expression of DLL4 in triple-negative breast cancer.**

**(a)** The statistical analysis of protein levels in Fig.4g. **(b)** The basal protein expression levels of NANOS1 in TNBC cell lines were detected by Western blot. **(c)** The protein expression levels of NANOS1 in NANOS1-vector/OE and scramble/shNANOS1 TNBC cell lines were detected by Western blot. **(d)** The statistical analysis of protein levels in Fig.4h. **(e)** The protein expression levels of NANOS1 and DLL4 in paired tumor (T) and non-tumor (N) tissues of TNBC patients were detected by Western blot. **(f)** Schematic illustration for the fat pad injection of 4T1-NANOS1-vector/OE and 4T1-scramble/shNANOS1 cell lines to C57BL/6 mice. **(g)** Tumor weight was analyzed in 4T1-NANOS1-vector and 4T1-NANOS1-OE groups. **(h)** Tumor weight was analyzed in 4T1-scramble and 4T1-shNANOS1 groups. **(i-j)** The protein expression levels of 4T1-NANOS1-vector, 4T1-NANOS1-OE, 4T1-scramble, and 4T1-shNANOS1 subcutaneous tumors of mice were detected by Western blot. Unpaired Student’s t-test was used for statistical analysis. **(k)** The statistical analysis of protein levels in Fig.4I. **(l)** The protein expression levels of DLL4 at different time points in NANOS1-vector/OE cell lines incubated with CHX were detected by Western blot. **(m)** Decay curves of DLL4 in indicated NANOS1-vector/OE cell lines incubated with CHX. **(n)** Detection of polyubiquitylated DLL4 protein by anti-Ub antibody. **(o-p)** The physical interaction between DLL4 and NANOS1 were verified by Co-IP assay. **(q-r)** The physical interaction between DLL4 and MIB1 in indicated NANOS1-vector/OE cell lines were verified by Co-IP assay. **(s)** Statistical analysis about binding of NANOS1 to the promoters of DLL4 determined by ChIP-qPCR. *P<0.05; **P<0.01; ***P<0.001; ****P<0.0001; ns, no significance.

**
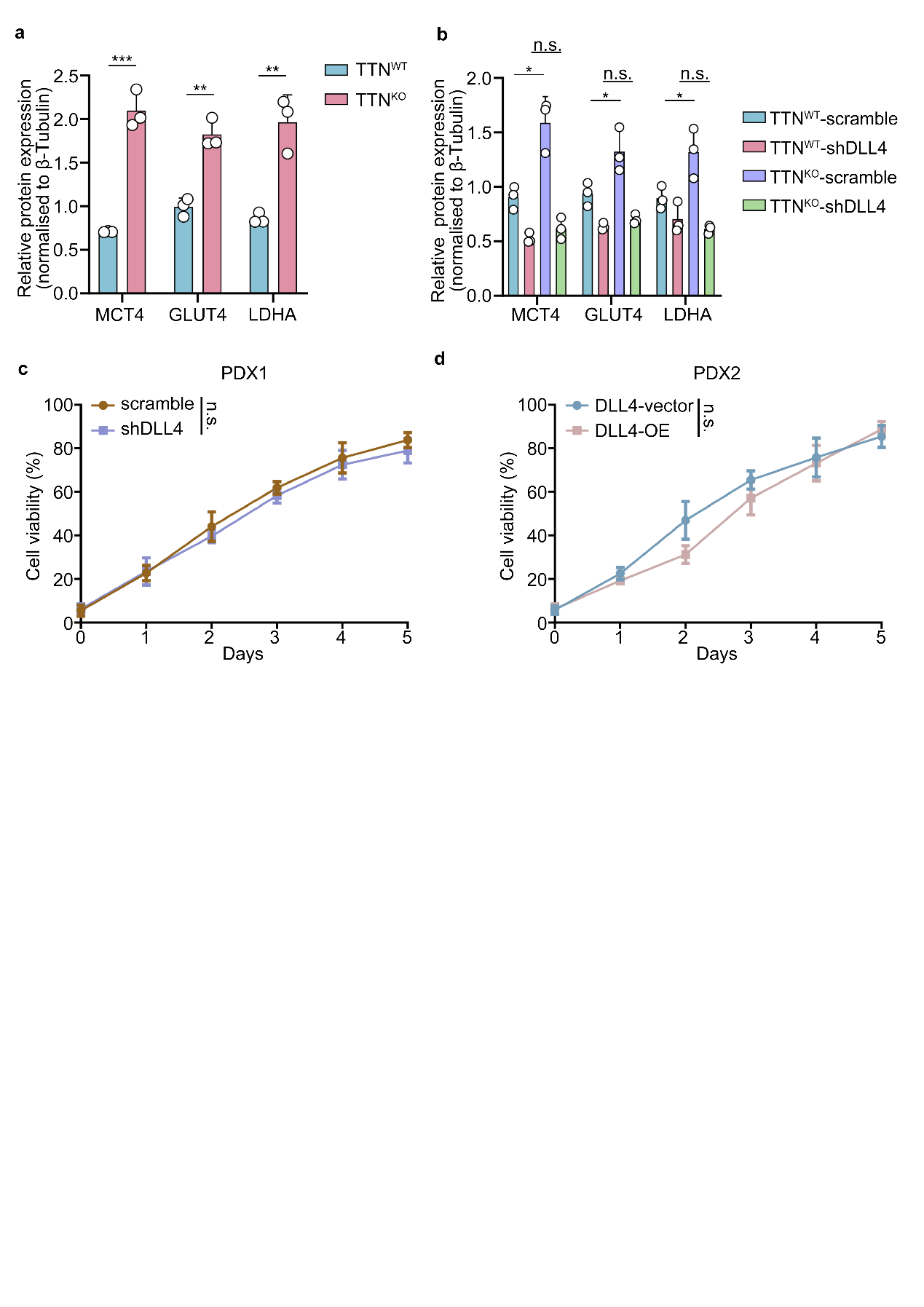
**

**Fig.S7. The cell viability curve of patient-derived tumor xenograft cells with the changes of DLL4 expression.**

**(a)** The statistical analysis of protein levels in Fig.5a. **(b)** The statistical analysis of protein levels in Fig.5k. **(a)** The cell viability curves of PDX1-scramble and PDX1-shDLL4 were detected by CCK-8 assay. **(b)** The cell viability curves of PDX2-DLL4-vector and PDX2-DLL4-OE were detected by CCK-8 assay. *P<0.05; **P<0.01; ***P<0.001; ns, no significance.


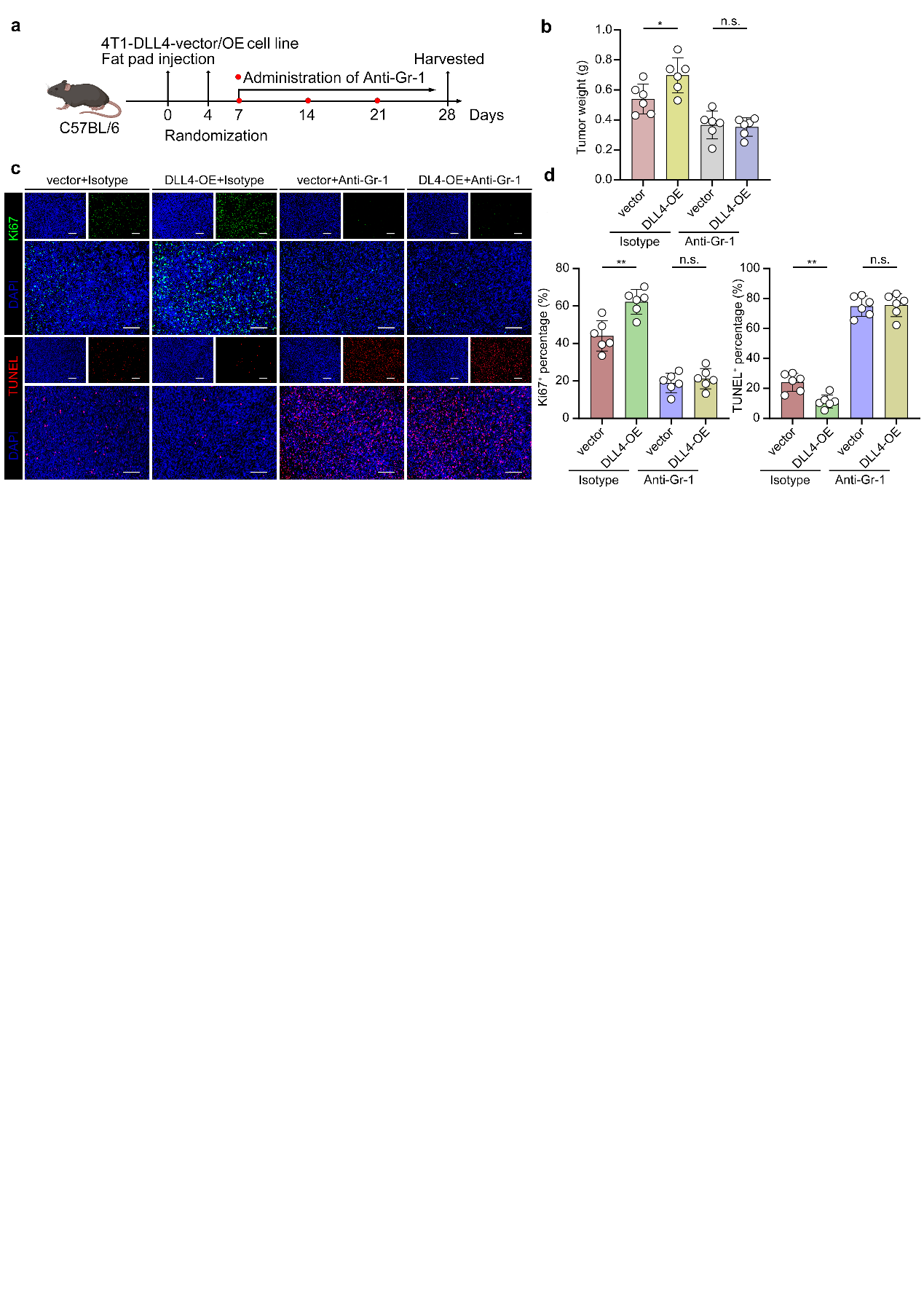


**Fig.S8. Myeloid-derived suppressor cells infiltration induced by DLL4 increased tumor burden in TTN-inactivation triple-negative breast cancer.**

**(a)** Schematic illustration for the fat pad injection of 4T1-DLL4-vector/OE cell lines to C57BL/6 mice with administration of Anti-Gr-1 or isotype. **(b)** Tumor weight was analyzed for each group. **(c-d)** The immunofluorescence staining of Ki67 and TUNEL of the subcutaneous tumor of each group was conducted. Representative images were shown **(c)**. The Ki67^+^ and TUNEL^+^ tumor cells were analyzed by Image-J software **(d)**. *P<0.05; **P<0.01; ns, no significance.


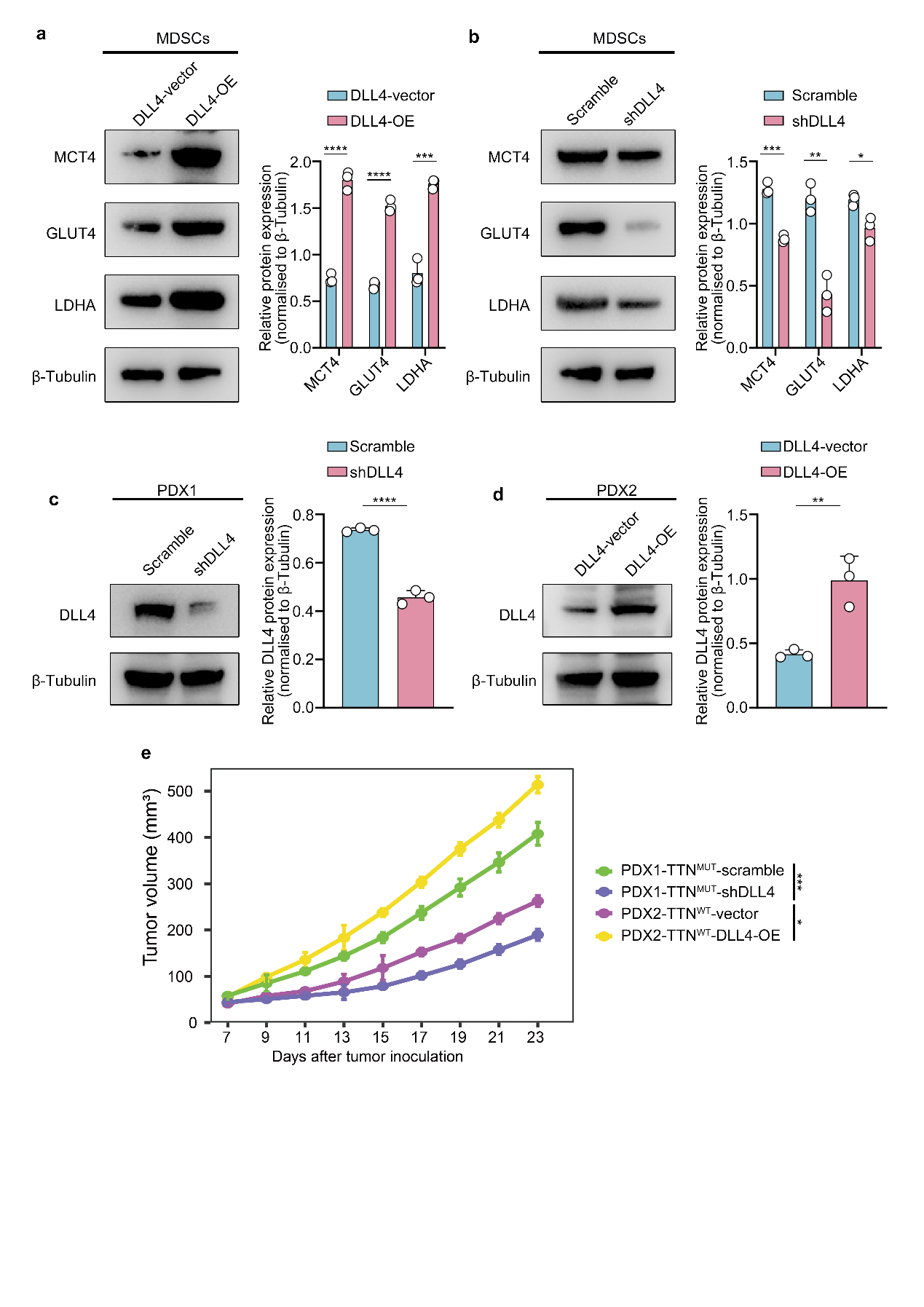


**Fig.S9. DLL4 induced the metabolic reprogramming of myeloid-derived suppressor cells in TTN inactivation triple-negative breast cancer tumors.**

**(a)** The protein expression levels of glycolytic markers of MDSCs in DLL4-vector and DLL4-OE groups were detected by Western blot. **(b)** The protein expression levels of glycolytic markers of MDSCs in the scramble and shDLL4 groups were detected by Western blot. **(c)** The protein expression levels of DLL4 in PDX1-scramble and PDX1-shDLL4 were detected by Western blot. **(d)** The protein expression levels of DLL4 in PDX2-DLL4-vector and PDX2-DLL4-OE were detected by Western blot. **(e)** The growth curve of PDX1-TTN^MUT^-scramble, PDX1-TTN^MUT^-shDLL4, PDX2-TTN^WT^-vector, and PDX2-TTN^WT^-DLL4-OE was plotted according to the size of the tumor. *P<0.05; ***P<0.001; ns, no significance. *P<0.05; **P<0.01; ***P<0.001; ****P<0.0001.


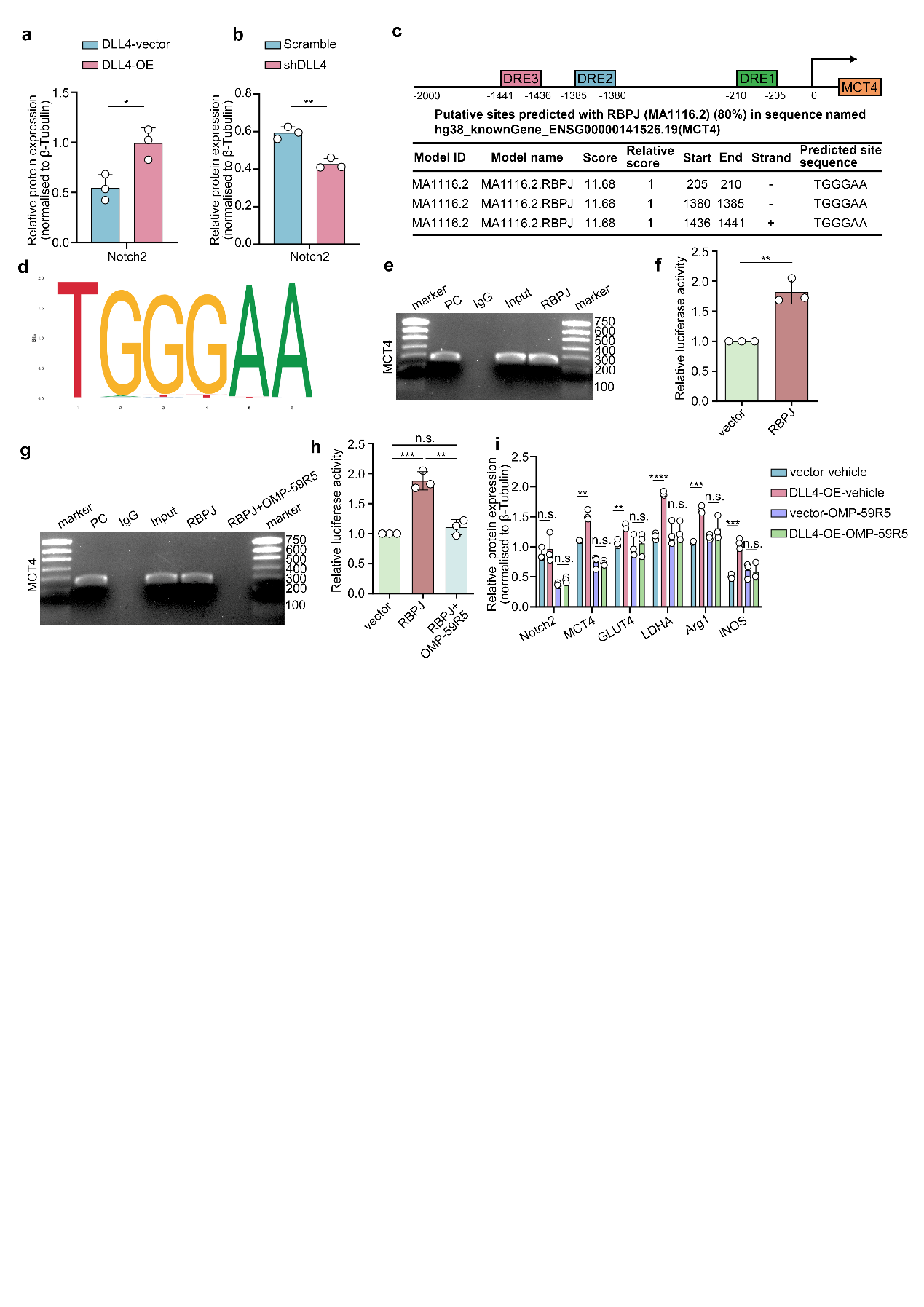


**Fig.S10. RBPJ promoted transcriptional regulation of MCT4 in myeloid-derived suppressor cells.**

**(a)** The statistical analysis of protein levels in Fig.6C. **(b)** The statistical analysis of protein levels in Fig.6D. **(c)** Predicted RBPJ binding sites in the human MCT4 promoters. Position relative to the transcription start site of the gene, sequence, and corresponding scores. **(d)** RBPJ-scanned motif logo. **(e)** ChIP assay was performed to validate the combination of RBPJ and MCT4 promoter. **(f)** The relative luciferase activity of the MCT4 promoter in the vector and RBPJ groups was measured by dual-luciferase assay. **(g)** ChIP assay was performed to validate the combination of RBPJ and MCT4 promoter after treatment with OMP-59R5. **(h)** The relative luciferase activity of the MCT4 promoter in the vector and RBPJ groups after treatment with OMP-59R5 was measured by dual-luciferase assay. **(i)** The statistical analysis of protein levels in Fig.6E. *P<0.05; **P<0.01; ***P<0.001; ****P<0.0001; ns, no significance.


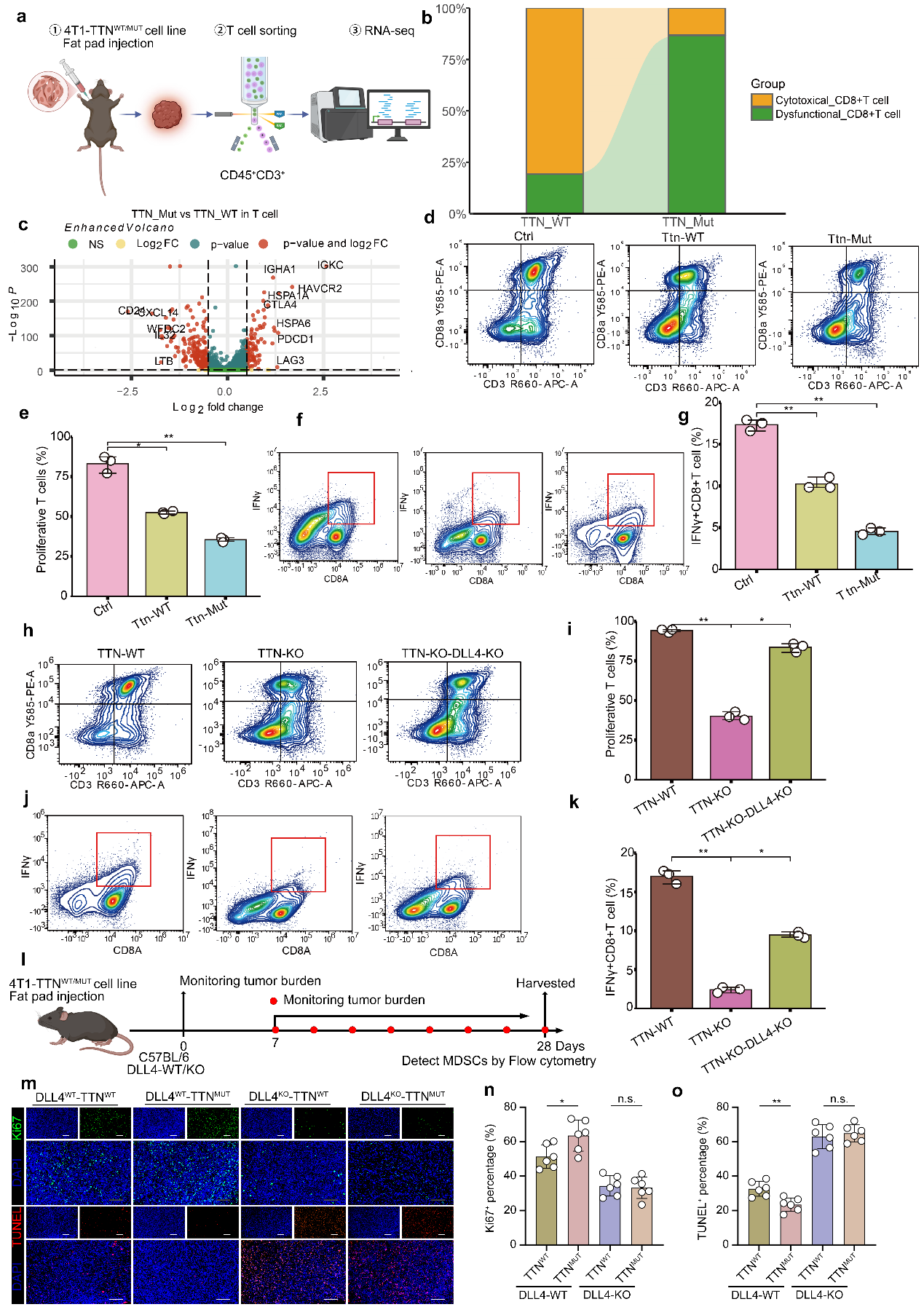


**Fig.S11. MDSCs induced by DLL4 inhibited the anti-tumor properties of T cells, thereby promoting tumor immune evasion.**

**(a)** Schematic illustration for sorted T cells RNA sequencing. **(b)** Volcano plot showing differentially expressed genes in the T cells of the TTN-WT and TTN-Mut groups. **(c)** The stacked bar chart showed the change trend of cytotoxic and dysfunctional CD8^+^ T cell subsets in TTN-WT and TTN-Mut groups. **(d-e)** The percentage of proliferative CD8^+^ T cells cocultured with MDSCs infiltrated into 4T1-TTN-WT and 4T1-TTN-Mut tumors determined by flow cytometry. **(f-g)** The percentage of IFNγ^+^CD8^+^ T cells cocultured with MDSCs infiltrated into 4T1-TTN-WT and 4T1-TTN-Mut tumors determined by flow cytometry. **(h-i)** The percentage of proliferative CD8^+^ T cells cocultured with MDSCs infiltrated into 4T1-TTN-WT, 4T1-TTN-KO, and 4T1-TTN-KO-DLL4-KO tumors determined based on flow cytometry. **(j-k)** The percentage of IFNγ^+^CD8^+^ T cells cocultured with MDSCs infiltrated into 4T1-TTN-WT, 4T1-TTN-KO, and 4T1-TTN-KO-DLL4-KO tumors determined based on flow cytometry. **(l)** Schematic illustration for the fat pad injection of 4T1-TTN-WT/MUT cell lines to C57BL/6-DLL4-WT or C57BL/6-DLL4-KO mice. **(m-o)** The immunofluorescence staining of Ki67 and TUNEL staining of the subcutaneous tumor in each group. Representative images **(m)** of Ki67^+^ **(n)** and TUNEL^+^ **(o)** tumor cells were analyzed by ImageJ software. Unpaired Student’s t-test was used for statistical analyses. *P<0.05; **P<0.01; ns, no significance.


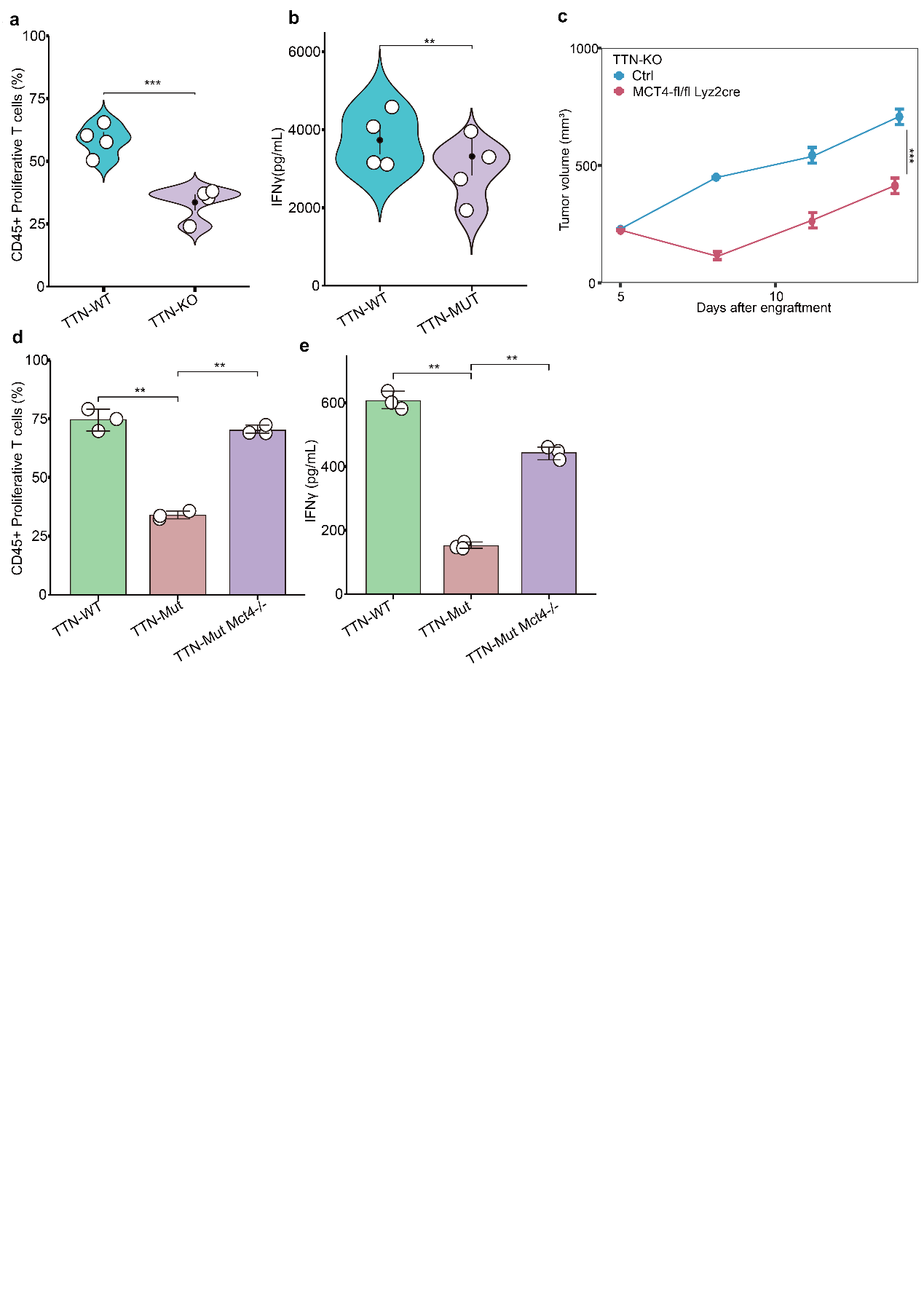


**Fig.S12. DLL4-NOTCH2-MCT4 axis suppressed T cell-mediated anti-tumor immunity.**

**(a)** The Percentage of proliferative T cells infiltrated in TTN-WT and TTN-KO tumors was determined by flow cytometry. **(b)** The quantitative contents of IFNγ in T cells cocultured with MDSCs infiltrated in 4T1-TTN-WT and 4T1-TTN-MUT tumors each group were detected by ELISA. **(c)** The growth curve of 4T1-TTN-KO fat pad injection tumors of MDSCs-MCT4^WT^ and MDSCs-MCT4^fl/fl^ mice was plotted according to the size of the tumor. **(d)** The percentage of proliferative CD8^+^ T cells in TTN-WT, TTN-MUT, and TTN-MUT-MCT4^fl/fl^ groups was determined by flow cytometry. **(e)** The percentage of IFNγ^+^CD8^+^ T cells in TTN-WT, TTN-MUT, and TTN-MUT-MCT4^fl/fl^ groups was determined by flow cytometry. Unpaired Student’s t-test was used for statistical analysis. **P<0.01; ***P<0.001.


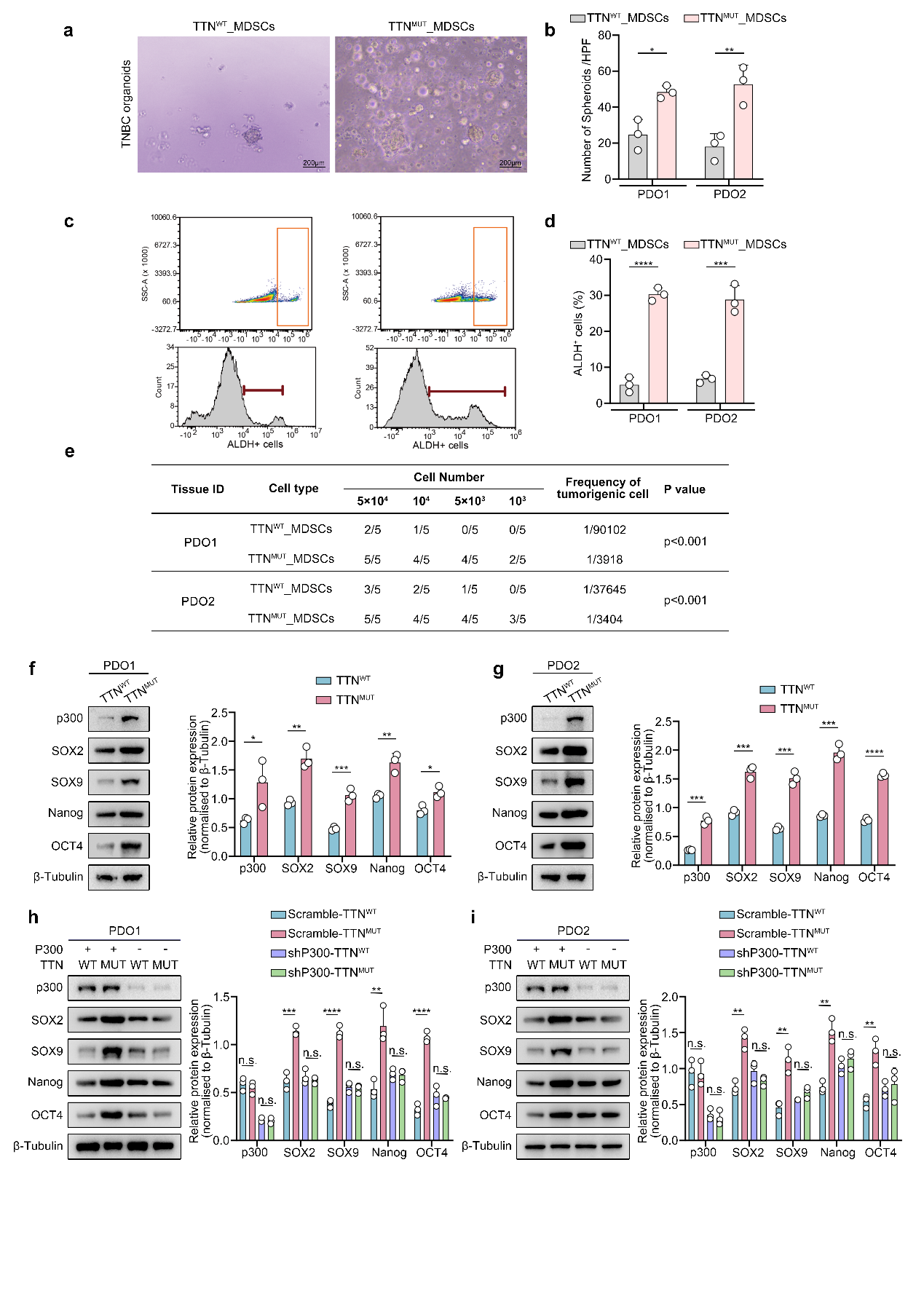


**Fig.S13. p300 was essential for H3K18la to enhance the stemness of triple-negative breast cancer tumors.**

**(a-b)** Sphere formation assays were performed in indicated groups of PDOs. **(c-d)** The percentage of ALDH^+^ cells in indicated groups of PDOs detected by flow cytometry. **(e)** In vivo limited dilution assays in indicated groups of PDOs. Representative tumor incidence and CSC probabilities are shown in the table. **(f)** The protein expression levels of p300 and stemness-related factors of the TTN-WT and TTN-Mut group in PDO1 were detected by Western blot. **(g)** The protein expression levels of p300 and stemness-related factors of the TTN-WT and TTN-Mut group in PDO2 were detected by Western blot. **(h)** The protein expression levels of stemness-related factors of the TTN-WT-scramble, TTN-MUT-scramble, TTN-WT-p300-KD, TTN-MUT-p300-KD of PDO1 were detected by Western blot. **(i)** The protein expression levels of stemness-related factors of the TTN-WT-scramble, TTN-MUT-scramble, TTN-WT-p300-KD, TTN-MUT-p300-KD of PDO1 were detected by Western blot. *P<0.05; **P<0.01; ***P<0.001; ****P<0.0001; ns, no significance.


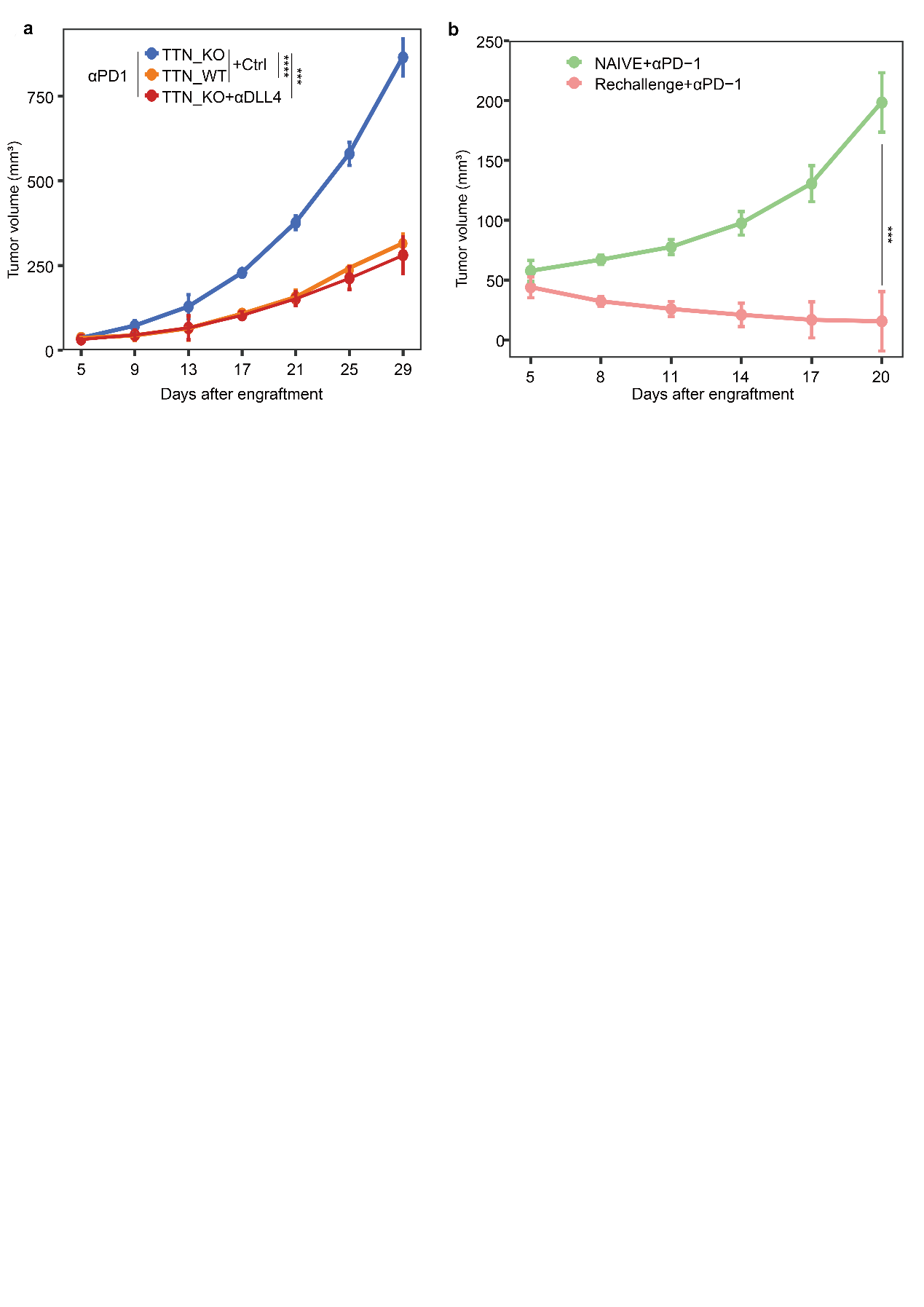


**Fig.S14. Blocking the TTN-DLL4-MCT4 axis could enhance the therapeutic effect of anti-PD1.**

**(a)** The growth curve of the TTN^KO^-αPD1, TTN^WT^-αPD1, and TTN^KO^-αDLL4+αPD1 groups was plotted according to the size of the tumor. **(b)** The growth curve of naive-αPD1 and rechallenge-αPD1 groups was plotted according to the size of the tumor. Unpaired Student’s t-test was used for statistical analysis. ***P<0.001; ****P<0.0001.


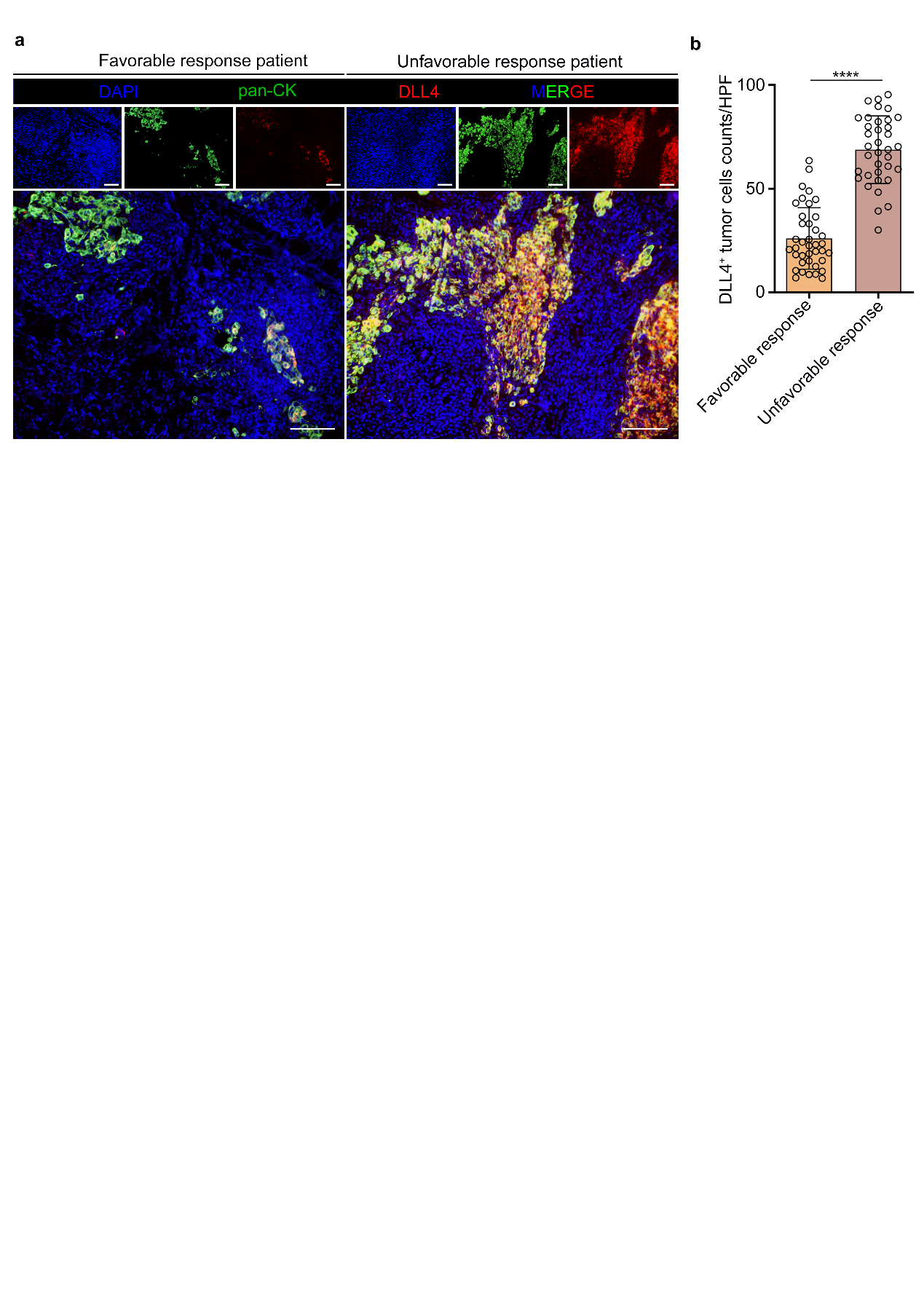


**Fig.S15. The DLL4 expression levels in neoadjuvant therapy favorable response and unfavorable response patients were detected by multiplex immunohistochemistry staining.**

**(a)** Representative images were shown. Bars, 100 µM. **(b)**The DLL4^+^ tumor cells were analyzed by Image-J software. n=75. ****P<0.0001.


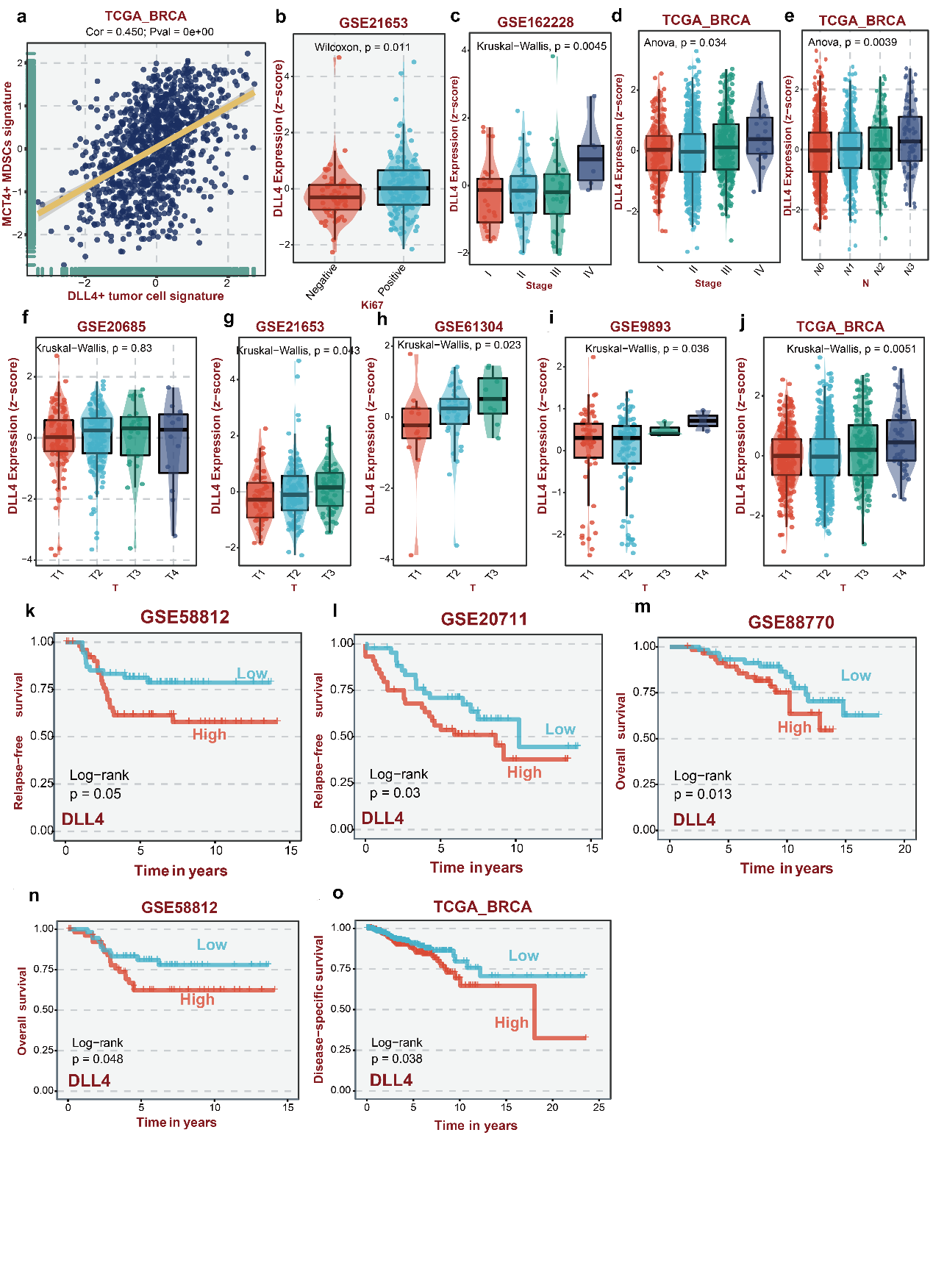


**Fig.S16. The DLL4 expression level could predict the adverse triple-negative breast cancer development and poor treatment response**

**(a)**Scatter plot showed the correlation between DLL4 expression and the proportion of MCT4^+^ MDSCs subpopulation **(b-j)** Boxplots illustrated the expression levels of DLL4 among various subgroups from different databases: between non-metastasis and metastasis groups **(b)**, among clinical stages **(c-d)**, among N stages **(e)** and T stages **(f-j)**. **(k-o)** KM curves plotted the relationship of DLL4 expression with the patients’ relapse-free survival **(k-l)**, overall survival **(m-n),** and disease-specific survival **(o)**from different databases.


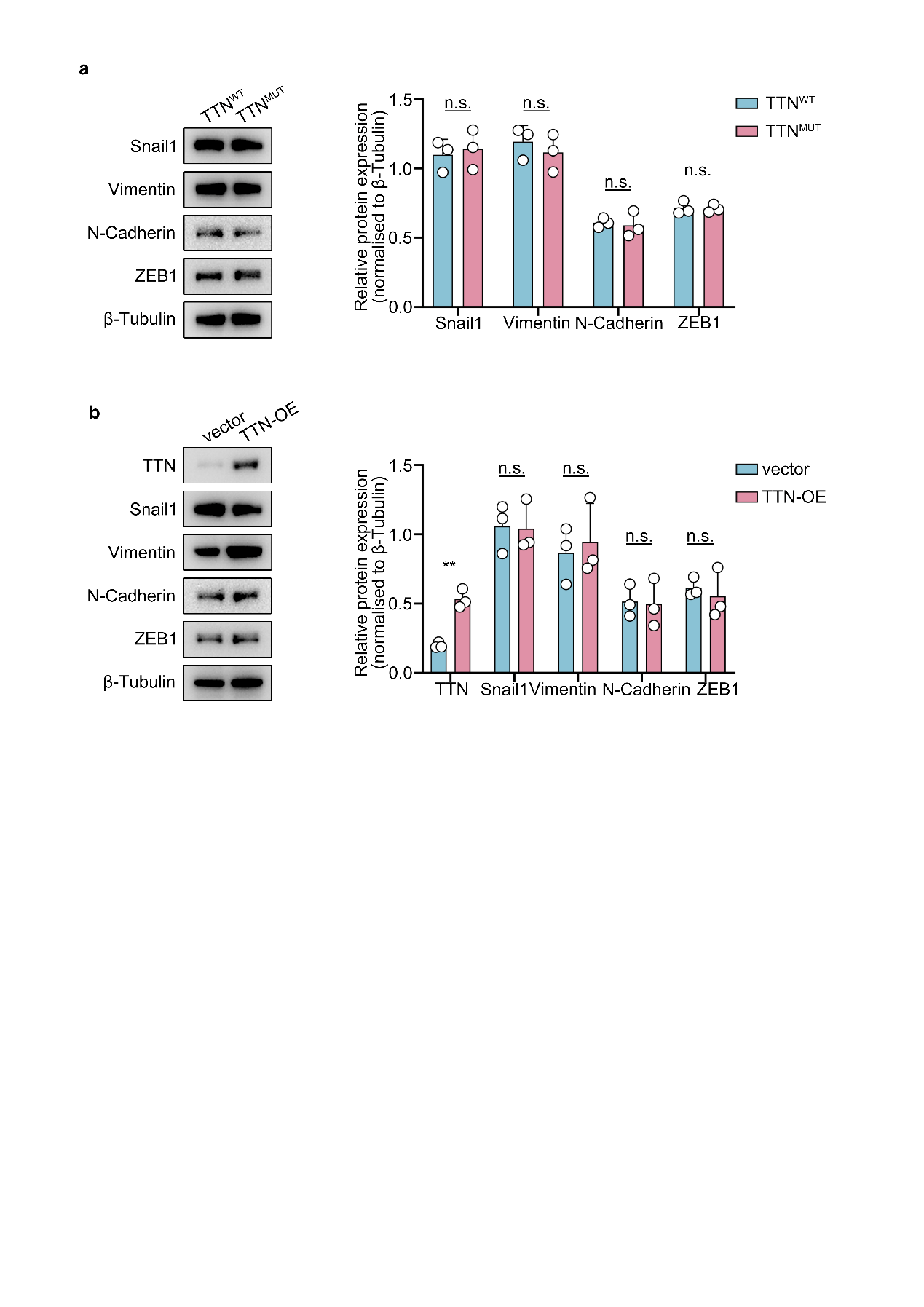


**Fig.S17. TTN inactivation could not affect the migration and metastasis phenotype of triple-negative breast cancer.**

**(a)** The protein expression levels of Snail1, Vimentin, E-cadherin and ZEB1 in TTN-WT and TTN-Mut MDA-MB-231 cells were detected by Western blot. **(b)** The protein expression levels of Snail1, Vimentin, E-cadherin and ZEB1 in TTN-vector and TTN-OE TNBC cells were detected by Western blot. **P<0.01; ns, no significance.

**Supplementary Tables**

**Table S1：Primers used for RT-PCR in this study**

| **Gene name** | **Forward** | **Reverse** |
| --- | --- | --- |
| DLL4 | CTGCGAGAAGAAAGTGGACAGG | ACAGTCGCTGACGTGGAGTTCA |
| MCT1 | TTGTTGGTGGCTGCTTGTCAGG | TCATGGTCAGAGCTGGATTCAAG |
| MCT4 | CCACAAGTTCTCCAGTGCCATTG | CGCCAGGATGAACACGTACATG |
| Arg1 | TCATCTGGGTGGATGCTCACAC | GAGAATCCTGGCACATCGGGAA |
| iNOS | GCTCTACACCTCCAATGTGACC | CTGCCGAGATTTGAGCCTCATG |
| SOX2 | GCTACAGCATGATGCAGGACCA | TCTGCGAGCTGGTCATGGAGTT |
| SOX9 | AGGAAGCTCGCGGACCAGTAC | GGTGGTCCTTCTTGTGCTGCAC |
| TTN | CTGCTGACTACACCTTTGTGGC | GCTCGCTTCTTCTCCAGTACCT |
| GAPDH | GTCTCCTCTGACTTCAACAGCG | ACCACCCTGTTGCTGTAGCCAA |

**Table S2：Antibodies used in this study**

| **Antibodies name** | **Dilution** | **Source** | **Cat#** | **RRID#** |
| --- | --- | --- | --- | --- |
| Anti-TTN Antibody (for IHC and WB) | 1:100 | Abcam | ab284862 | AB_2915962 |
| Anti-panCK Antibody (for mIHC) | 1:100 | Abcam | ab7753 | AB_306047 |
| Anti-CD15 Antibody (for mIHC) | 1:100 | Abcam | ab135377 | AB_3662850 |
| Anti-Arg1 Antibody (for WB) | 1:1000 | CST | 93668 | AB_2800207 |
| Anti-iNOS Antibody (for WB) | 1:1000 | Abcam | ab178945 | AB_2861417 |
| Anti-DLL4 Antibody (for IHC and WB) | 1:100 | Abcam | ab183532 | AB_2747593 |
| [Anti-Mib1/Mindbomb](https://www.abcam.cn/products/primary-antibodies/mib1-mindbomb-antibody-ab74134) Antibody (for WB) | 1:1000 | Abcam | ab74134 | AB_1269426 |
| Anti-KAT3B / p300 Antibody (for WB) | 1:1000 | Abcam | ab10485 | AB_297224 |
| Anti-SOX2 Antibody (for WB) | 1:1000 | Abcam | ab92494 | AB_10585428 |
| Anti-SOX9 Antibody (for WB) | 1:1000 | Abcam | ab185966 | AB_2728660 |
| Anti-Nanog Antibody (for WB) | 1:1000 | Abcam | ab109250 | AB_10863442 |
| Anti-Oct4 antibody (for WB) | 1:1000 | Abcam | ab109183 | AB_10864777 |
| Anti-SNAIL antibody (for WB) | 1:1000 | Abcam | ab31787 | AB_1281121 |
| Anti-Vimentin antibody (for WB) | 1:1000 | Abcam | ab92547 | AB_10562134 |
| Anti-N Cadherin antibody (for WB) | 1:1000 | Abcam | ab76011 | AB_1310479 |
| Anti-ZEB1 antibody | 1:1000 | Abcam | ab87280 | AB_2040541 |
| Anti-EpCAM Antibody (for mIHC) | 1:100 | Abcam | ab223582 | AB_2762366 |
| BV421 anti-mouse CD45RA Antibody (for FCM) | 5μL/test | BD | 740022 | AB_2739794 |
| PE-Cy7 anti-mouse CD11b Antibody (for FCM) | 5μL/test | BD | 561098 | AB_2033994 |
| APC anti-mouse Ly6G Antibody | 5μL/test | BD | 560599 | AB_1727560 |
| FITC anti-mouse Ly6C Antibody | 5μL/test | BD | 553104 | AB_394628 |
| APC anti-mouse Gr-1 Antibody (for FCM) | 5μL/test | BD | 553129 | AB_398532 |
| Anti-NANOS1Antibody (for IHC and WB) | 1:100 | Invitrogen | PA5-20556 | AB_11155848 |
| Anti-Cytokeratin 19 Antibody (for WB) | 1:100 | Abcam | ab76539 | AB_1523469 |
| Anti-MCT4 antibody (for WB) | 1:1000 | Santa Cruz | sc-376465 | AB_11150118 |
| Anti-GLUT4 antibody (for WB) | 1:1000 | Santa Cruz | sc-53566 | AB_629533 |
| Anti-LDHA antibody (for WB) | 1:1000 | Santa Cruz | sc-137244 | AB_2250008 |
| Anti-Noth2 antibody (for WB) | 1:1000 | Abcam | ab307700 | AB_2267338 |
| RB705 anti-human CD45 Antibody (for FCM) | 5μL/test | BD | 757307 | AB_3689469 |
| PE anti-human CD11b Antibody (for FCM) | 5μL/test | BD | 555388 | AB_395789 |
| FITC anti- human CD14 Antibody | 5μL/test | BD | 561712 | AB_395798 |
| APC anti-human CD15 Antibody (for FCM) | 5μL/test | BD | 561716 | AB_10893192 |
| CoraLite® Plus 488 anti-human MCT4 Antibody (for FCM) | 5μL/test | proteintech | CL488-22787 | AB_3084070 |
| PE anti-mouse CD206 Antibody (for FCM) | 5μL/test | BD | 568273 | AB_2916867 |
| APC anti-mouse CD3e Antibody (for FCM) | 5μL/test | BD | 561826 | AB_10896663 |
| PE anti-mouse CD8αAntibody (for FCM) | 5μL/test | BD | 567630 | AB_2916674 |
| PE-Cy7 anti-mouse IFNγ Antibody (for FCM) | 5μL/test | BD | 561040 | AB_2034014 |
| Anti-Ki67 Antibody (for WB) | 1:100 | abcam | ab264429 | AB_3674140 |
| PE anti-mouse CD44 Antibody (for FCM) | 5μL/test | BD | 561860 | AB_10895375 |
| APC anti-mouse CD24 Antibody (for FCM) | 5μL/test | BD | 567807 | AB_3683922 |
| Anti-β-Tubulin Antibody (for WB) | 1:5000 | Beijing Ray Antibody Biotech | RM2003L | AB_2773015 |
| Anti-mouse CD3ε antibody | 5 μg/mL | Biolegend | 100339 | AB_11150783 |
| Anti-mouse CD28 antibody | 5 μg/mL | Biolegend | 102115 | AB_11150408 |
